# Interaction profiling of RNA-binding ubiquitin ligases reveals a link between posttranscriptional regulation and the ubiquitin system

**DOI:** 10.1038/s41598-017-16695-6

**Published:** 2017-11-29

**Authors:** Andrea Hildebrandt, Gregorio Alanis-Lobato, Andrea Voigt, Kathi Zarnack, Miguel A. Andrade-Navarro, Petra Beli, Julian König

**Affiliations:** 1 Institute of Molecular Biology (IMB), Ackermannweg 4, 55128 Mainz, Germany; 2 Faculty of Biology, Johannes Gutenberg University, Gresemundweg 2, 55128 Mainz, Germany; 3 Buchmann Institute for Molecular Life Sciences (BMLS), Goethe University Frankfurt, Max-von-Laue-Str. 15, 60438 Frankfurt, Germany

## Abstract

RNA-binding ubiquitin ligases (RBULs) have the potential to link RNA-mediated mechanisms to protein ubiquitylation. Despite this, the cellular functions, substrates and interaction partners of most RBULs remain poorly characterized. Affinity purification (AP) combined with quantitative mass spectrometry (MS)-based proteomics is a powerful approach for analyzing protein functions. Mapping the physiological interaction partners of RNA-binding proteins has been hampered by their intrinsic properties, in particular the existence of low-complexity regions, which are prone to engage in non-physiological interactions. Here, we used an adapted AP approach to identify the interaction partners of human RBULs harboring different RNA-binding domains. To increase the likelihood of recovering physiological interactions, we combined control and bait-expressing cells prior to lysis. In this setup, only stable interactions that were originally present in the cell will be identified. We exploit gene function similarity between the bait proteins and their interactors to benchmark our approach in its ability to recover physiological interactions. We reveal that RBULs engage in stable interactions with RNA-binding proteins involved in different steps of RNA metabolism as well as with components of the ubiquitin conjugation machinery and ubiquitin-binding proteins. Our results thus demonstrate their capacity to link posttranscriptional regulation with the ubiquitin system.

## Introduction

Posttranscriptional RNA processing provides a fundamental step in the regulation of gene expression. RNA-binding proteins (RBPs) play a key role in posttranscriptional regulation by determining the fate and function of their target transcripts. Recent large-scale mRNA interaction profiling studies have demonstrated that eukaryotic cells express >1,200 RBPs^1–3^. In addition to their RNA binding capability, many of these RBPs possess catalytic activities, thus introducing an additional layer of complexity into posttranscriptional regulation^1,3^. A particularly interesting class of proteins in this context are RNA-binding ubiquitin ligases (RBULs) that contain either a HECT, RING or ring between ring (RBR) ubiquitin ligase domain. The human genome encodes at least 26 putative RING-type RBULs, which can be assigned to five different families according to their RNA-binding domains (RBDs), including CCCH zinc fingers, K-homology (KH) domains, RNA recognition motifs (RRM) and KKKTK (K-rich) regions^4^. The existence of the RNA-binding and ubiquitin ligase functions in a single protein has the potential to link posttranscriptional regulation of gene expression with the ubiquitin system. Despite this, only a few RBULs have been characterized to date and most previous studies focused on a specific function of a single RBUL.

Mapping of protein interaction partners is frequently employed to analyze cellular functions of poorly characterized proteins. To this end, a bait protein and its interaction partners are purified by antibodies or affinity resins, and the interaction partners are identified by liquid chromatography-tandem mass spectrometry (LC-MS/MS). This approach allows to efficiently discriminate physiological interactions from background binders, when combined with quantitative MS approaches such as stable isotope labeling with amino acids in cell culture (SILAC)^5^. However, several characteristics of RBPs impede the identification of physiological interactions in proteomics approaches. RBPs often contain disordered regions, including repetitive sequences and low-complexity domains, which can promote dynamic interactions with other proteins even in cell lysates^3,6^. Native ribonucleoprotein complexes were previously shown to extensively reassemble already during cell lysis, thereby concealing the interaction landscape of the native cell^7^. In addition, several RBPs were recently described to harbor the ability to transition into a hydrogel-like state that likely facilitates non-physiological protein associations^8^. Taken together, the above-mentioned properties of RBPs interfere with a specific enrichment of physiological interactions through affinity purification (AP) and create a demand for well-adapted sample preparation strategies.

**Figure 1:**
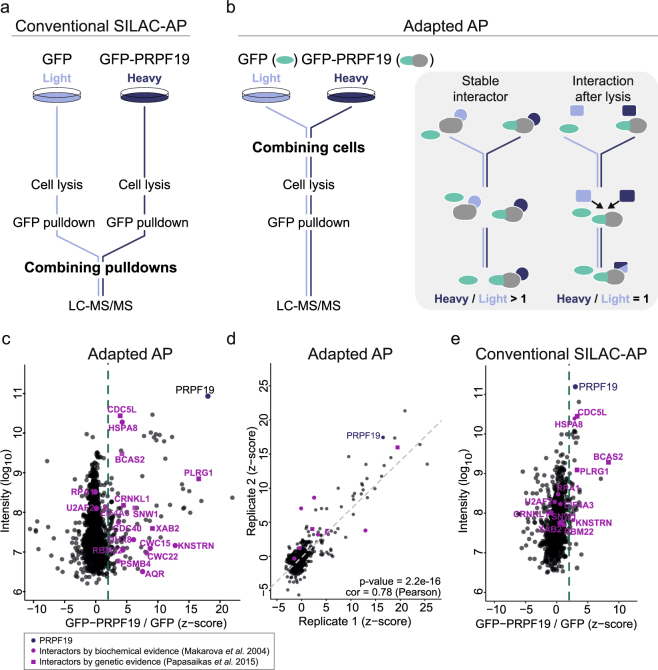
The adapted SILAC-based AP approach efficiently recovers known interactors of PRPF19. (**a**) In the conventional SILAC-AP, GFP alone is expressed in light-isotope labeled cells (light blue), while GFP-tagged PRPF19 is expressed in heavy isotope-labeled cells (dark blue). Upon cell lysis, GFP/GFP-tagged bait proteins are subjected to affinity purification (AP) with GFP-trap agarose beads. Enriched proteins are mixed in a 1:1 ratio and analyzed by LC-MS/MS. (**b**) For the adapted AP protocol, differentially isotope-labeled cells are mixed at equal amounts and lysed as a pool. Upon AP and on-bead digest with trypsin, enriched proteins are analyzed by LC-MS/MS. In this setup, only stable interactions that were already present *in vivo* will obtain high SILAC ratios (H/L), whereas transient interactors and any associations formed after cell lysis will display equalized label occurrences. (**c**) The adapted AP for GFP-PRPF19 identifies 13 previously known PRPF19 interactors. Shown are the SILAC ratios of one individual biological replicate after z-score normalization plotted against log_10_ transformed intensities. PRPF19 is labeled in dark blue. Previously described PRPF19 interactors that were biochemically shown to interact^15^ or assigned by genetic evidence^72^ are displayed by round or square purple symbols, respectively. The applied cut-off at z-score ≥ 2 is labeled by a dashed line. (**d**) Comparison of two independent biological replicates of the adapted AP with GFP-PRPF19. Visualization and labeling as in (**c**). A linear regression line is indicated by a grey dashed line. (**e**) Pulldowns of GFP-PRPF19 following the conventional SILAC-AP protocol. SILAC ratios (after z-score normalization) are plotted against log_10_ transformed intensities of one biological replicate. Visualization and labeling as in (**c**).

Depending on the stability of the studied interactions, different sample preparation strategies have been developed. For instance, the conventional SILAC-AP workflow efficiently captures both stable and transient interactions by separately processing all samples until after AP (Fig. 1a). In contrast, purification after mixing (PAM)-SILAC employs combining of differentially isotope-labeled cell lysates prior to AP^9^. In this setup, transient interactors with high on/off rates quickly reach an equilibrium of light and heavy labeled forms, resulting in SILAC ratios close to background levels. Conversely, stable associations that are maintained throughout purification retain high SILAC ratios. However, neither of the two sample preparation strategies is immune to proteins that engage in non-physiological interactions already during cell lysis. Although not occurring under physiological conditions, such newly formed protein associations might display high stability, for instance if high-affinity binders had been precluded from each other *in vivo* by cellular compartmentalization. Here, we present an adapted SILAC-based AP approach (referred to adapted AP) that employs mixing of equal amounts of differentially isotope-labeled cells prior to the cell lysis (Fig. 1b). Because light and heavy isotope-labeled forms of all proteins are mixed during lysis, newly established interactions will display low SILAC ratios irrespective of their biophysical properties. Consequently, the described approach specifically recovers stable interactions that were present within the intact cell and is complementary to other approaches, such as the conventional SILAC-AP, tandem affinity purifications (TAP) and biotin-based proximity tagging (APEX and BioID)^10–12^. We benchmark our approach in its ability to recover physiological interactions using Gene Ontology (GO) similarity measures to score the coherence of functional annotations between the bait and its putative interaction partners. Employing the described approach, we identify the interaction partners of six human RBULs (ARIH2, MEX3B, MKRN1, MKRN2, RNF17 and PRPF19). In addition to at least one RING E3 ubiquitin ligase domain, the selected RBULs harbor different RBDs, such as WD repeats (PRPF19), KH domains (MEX3B), Tudor domains (RNF17) or CCCH zinc fingers (MKRN1/2). We further include ARIH2, which does not show an obvious RBD according to current predictions, but was previously found in association with the nuclear polyA-binding protein PABPN1^13^.

**Figure 2:**
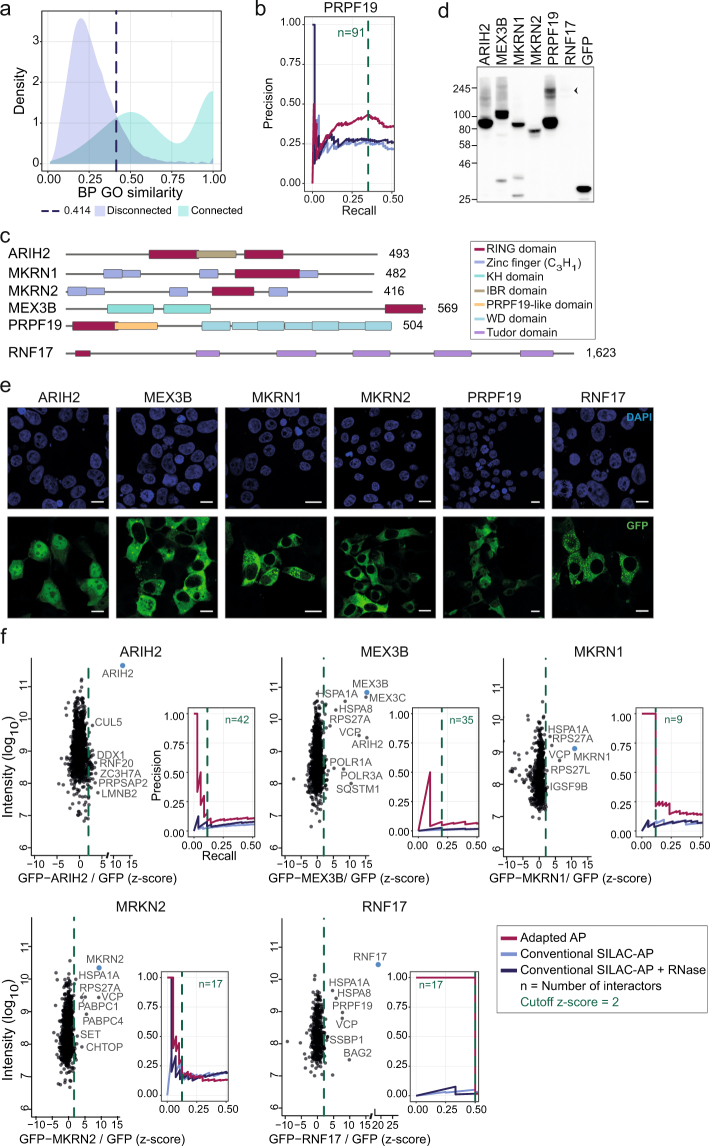
Experiments following the adapted AP approach and GO similarity analyses for six RBULs. (**a**) Benchmarking of the GO similarity strategy by comparing the WI distribution of interacting protein pairs from the INstruct database (turquoise) to the control set of disconnected protein pairs (lilac). Applying a 10% FDR, a ‘true positive’ interactor is defined by a WI ≥ 0.414 (dashed blue line). (**b**) Precision-Recall curves of the conventional SILAC-AP data without (blue) or with RNase treatment (dark blue), and the adapted AP data (red) for GFP-PRPF19. Detected proteins were ranked according to their SILAC ratios, and precision and recall of GO evidence-supported interactors were calculated based on the similarity of BP GO terms between bait and prey proteins (WI ≥ 0.414). The dashed green line indicates applied cut-off at z-score ≥ 2. n, number of PRPF19 interactors. Legend shown in (**f**). (**c**) Predicted domain architecture of the six tested RBULs. Domains are indicated on the right. (**d**) Expression of GFP-RBULs measured by Western blot. GFP-tagged RBULs were transiently expressed in HEK293T cells, and their expression analyzed by Western blot. A representative experiment of two biological replicates is shown. GFP-RNF17 is highlighted by an arrowhead; a longer exposure is shown in Supplementary Figure 3c. All GFP-tagged RBULs are detected at the expected size. (**e**) Subcellular localization of GFP-tagged RBULs. GFP-tagged RBULs were expressed in HEK293T cells and their subcellular localization was analyzed by confocal microscopy. Scale bars indicate 10 µm. (**f**) Ratio-intensity plots of adapted AP experiments and Precision-Recall curves for the RBULs ARIH2, MEX3B, MKRN1, MKRN2, and RNF17 are shown. Combined SILAC ratios from three independent biological replicates are plotted against log_10_ transformed intensities. Bait proteins (blue) and selected preys with high SILAC ratios are indicated.

For all investigated RBULs, our analyses highlight their extensive involvement in ubiquitin-mediated functions, illustrated for instance by the interaction of five RBULs with the ubiquitin-dependent co-chaperone VCP. In addition, we find evidence that the studied RBULs are involved in different posttranscriptional pathways, including translational regulation, ribonucleoprotein assembly and splicing. The benchmarking of our approach using GO similarity testing further supports the notion that combining cells prior to lysis increases the likelihood of recovering stable physiological interactions when studying RBPs.

## Results

### Strategy for recovering stable interaction partners of RNA-binding ubiquitin ligases

In a conventional SILAC-based AP experiment, the bait and control pulldowns are performed separately and the enriched proteins are combined after the washing steps. This setup enables the quantitative comparison of different conditions and minimizes the intra-sample variance^14^. However, we reasoned that this approach might be suboptimal when analyzing RBPs, which are prone to form non-physiological interactions in the cell lysate. We therefore employed an adapted AP that disfavors dynamically exchanging or newly established protein interactions after cell lysis to analyze the stable interaction partners of RBULs. To this end, heavy isotope-labeled cells expressing the GFP-tagged bait are combined already prior to cell lysis with light isotope-labeled control cells expressing GFP only (Fig. 1b). Interaction partners with high exchange rates will display both labels with equal probability, resulting in low, close-to-background SILAC ratios for these transient interactions. Reversely, stable physiological interaction partners that associated with the bait protein in the cell will remain constantly bound during the sample preparation, thus displaying high SILAC ratios (Fig. 1b).

**Figure 3:**
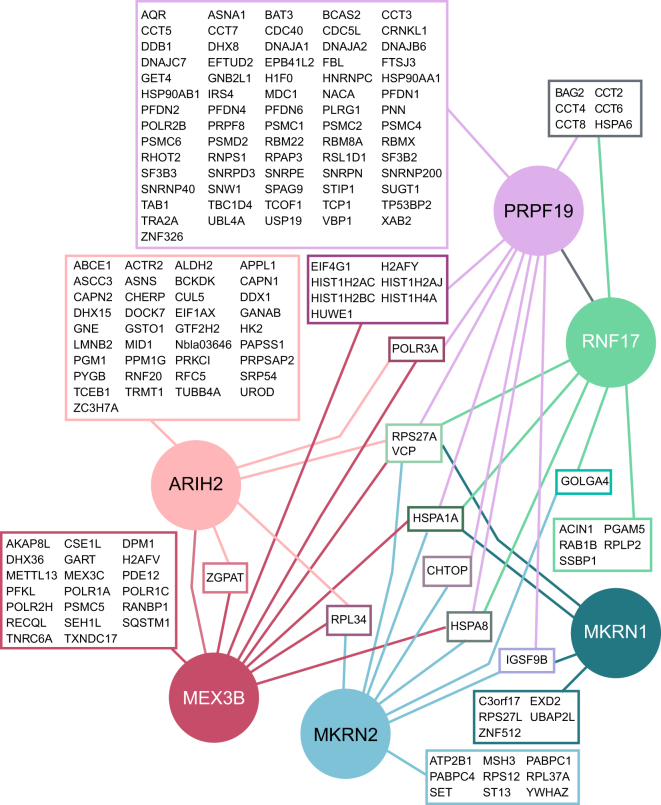
The core interactomes of six RBULs. The core interactomes of the six RBULs ARIH2, MEX3B, MKRN1, MKRN2, RNF17 and PRPF19 were identified using the adapted AP. Baits are shown in filled circles, and preys in framed boxes.

In order to test the performance of our adapted AP, we analyzed its ability to identify the interaction partners of the well-characterized RBUL PRPF19. PRPF19 is part of the PRP19/CDC5L complex (also known as Nineteen complex [NTC]) and the XAB2 complex and plays a role in the catalytic activation of the spliceosome^15–18^. GFP-tagged PRPF19 was expressed in human HEK293T cells. Western blot experiments with a PRPF19-specific antibody that detects both endogenous and GFP-tagged PRPF19 suggested a 37-fold lower expression compared to the endogenous counterpart (Suppl. Figure 3a). Apart from the previously described nuclear localization (Human Protein Atlas^19^, http://www.proteinatlas.org/), we found a fraction of GFP-PRPF19 also in the cytoplasm (Fig. 2e, Suppl. Table 6). For isotopic labeling, GFP-PRPF19-expressing cells were grown in medium containing heavy isotope-labeled arginine and lysine, while empty vector-expressing cells grown in medium containing light isotope-labeled arginine and lysine served as control. Equal numbers of light and heavy isotope-labeled cells were combined, followed by lysis and AP using GFP-Trap agarose beads. Immunoprecipitated proteins were digested on-bead with trypsin, and peptides were identified by LC-MS/MS^20^. In total, we quantified 1,061 protein groups in at least two replicate experiments (at least two peptides, including one unique peptide, and two ratio counts; Suppl. Table 3). The quantified SILAC ratios were log_2_ transformed and converted into z-scores^21,22^. We confirmed the reproducibility of our measurements by comparing independent experimental replicates (Fig. 1d). All proteins with an average z-score ≥ 2 across five replicate experiments, meaning that they differ by at least two standard deviations from the mean of the positive values in the distribution, were considered as putative interaction partners (dashed line, Fig. 1c). Out of 1,061 quantified proteins, 91 proteins displayed a z-score ≥ 2 in the GFP-PRPF19 pulldown compared to control, thus representing putative stable interaction partners of PRPF19 (Figs 1c and 3, Suppl. Tables 1–3). Notably, 17 out of these 91 (19%) proteins have been previously described as PRPF19 interaction partners according to the Human Integrated Protein-Protein Interaction Reference (HIPPIE) database^23^ (Suppl. Table 5). Moreover, 13 of these represent well known PRPF19 interactors, including the complete NTC core module (CDC5L, PLRG1, and BCAS2) as well as XAB2 and AQR from the XAB2 complex^15,16^ (Fig. 1c). To validate the performance and specificity of our approach, we performed a conventional SILAC-AP for GFP-PRPF19, in which samples are combined after AP. In order to discriminate RNA-mediated interactions, we included an additional condition, in which the samples were subjected to optimized RNase A/RNase T1 digest after the AP (Suppl. Figures 1 and 2a). We quantified 632 protein groups, out of which 16 had a z-score ≥ 2 and were thus regarded as putative interaction partners of GFP-PRPF19 (Suppl. Tables 1 and 3). Among these, only one known interaction partner was recovered (compared to 13 in the pulldown applying the adapted AP) (Fig. 1e). 5 of 16 proteins were lost after RNase digestion, indicating that RNA-mediated protein interactions account for a minor fraction of detected interactions when analyzing RBPs (Suppl. Figure 2b). In summary, we show that the adapted AP based on combining proteins already at the cell level enabled us to substantially increase the recovery rate of known interaction partners compared to the conventional SILAC-AP. These results underline the increased performance and specificity of the adapted AP approach in analyzing the interaction partners of RBPs.

### Functional coherence of the identified interaction profile of PRPF19

As outlined above, the adapted AP is suited to capture stable interaction partners, whereas loosely associated proteins will be categorized as background binders. We postulated that physiological interaction partners are more likely to participate in the same cellular processes as the bait protein compared to spuriously associated proteins. We therefore investigated the similarity of the associated Gene Ontology (GO) terms^24^. To this end, we computed the Wang’s Index (WI) as a measure of pair-wise similarity of Biological Process (BP) GO terms between the bait and the putative interaction partners identified by the adapted AP^25,26^. To benchmark this approach, we extracted known high-quality protein interactions from the structurally resolved INstruct network and compared the WI distributions of connected and disconnected protein pairs^27,28^. This analysis showed that WIs of connected proteins are significantly higher than those of disconnected pairs (*P* value < 2.2 × 10^−16^, Mann-Whitney U test; Fig. 2a), confirming that they offer meaningful evidence for true interactions. Based on the WI distribution for disconnected pairs, we estimated an empirical false discovery rate (FDR) and defined a cut-off at FDR <10% (corresponding to a WI = 0.414; Fig. 2a). Under this premise, we consider a RBUL-interactor pair as ‘true’ (i.e. supported by GO evidence), if the associated GO-BP terms yield a WI ≥ 0.414, enabling us to compile a reference set of ‘true-positive’ functionally coherent interactions. This method thus enables us to evaluate potential protein-protein interactions, identified by the adapted AP, for their probability to interact physiologically.

Based on the GO evidence, we compared the performance of the adapted AP with the conventional SILAC-AP based on Precision-Recall curves (Fig. 2b). To this end, we ordered the RBUL-interactor pairs by decreasing SILAC ratios and iteratively considered an increasing number of interaction pairs from the top of the list. For each set of interaction pairs, we computed the fraction of functionally coherent interactions (WI ≥ 0.414) with respect to the current set (precision) and the full list (recall). Putative PRPF19 interactors determined by the adapted AP showed strong signal enrichment, with increased precision associated with higher SILAC ratios. As expected, precision is sacrificed beyond a certain level of sensitivity (recall) when unspecific interactions begin to prevail. Notably, the adapted AP performs considerably better than the conventional SILAC-AP, underlining its ability to identify physiological interaction partners of RBPs. In line with the notion that some unspecific interactions could be mediated by RNA, the additional RNase digest slightly improved the performance in the GO similarity scoring. In summary, our adapted AP in combination with computational evaluation based on GO similarities offers a useful approach to identify stable protein interactions of RBPs and can be used complementary to the conventional SILAC-AP. We benchmarked this approach on the example of PRPF19, which enabled the identification of previously known as well as novel interaction partners of this RBUL.

### Defining the interaction profiles of selected RBULs

Reassured by the high efficiency in recovering known protein interaction partners of PRPF19, we applied our adapted AP and the computational evaluation steps to further RBULs. We selected MEX3B, MKRN1, MKRN2 and RNF17, which contain different RBDs and represent different RBUL families (Fig. 2c). We additionally included ARIH2, which does not harbor an RBD but was previously shown to interact with the nuclear polyA-binding protein PABPN1^13^. We first used fluorescence microscopy to confirm the subcellular localization of the tagged proteins (Fig. 2e). All GFP-tagged RBULs localized to the expected compartments as reported in the Human Protein Atlas^19^ and previous publications (Fig. 2e, Suppl. Table 6). For MEX3B, we additionally performed Western blots with a specific antibody that detects both endogenous and GFP-tagged MEX3B, estimating a 13-fold increased expression level of the tagged version compared to the endogenous counterpart (Suppl. Figure 3a).

**Figure 4:**
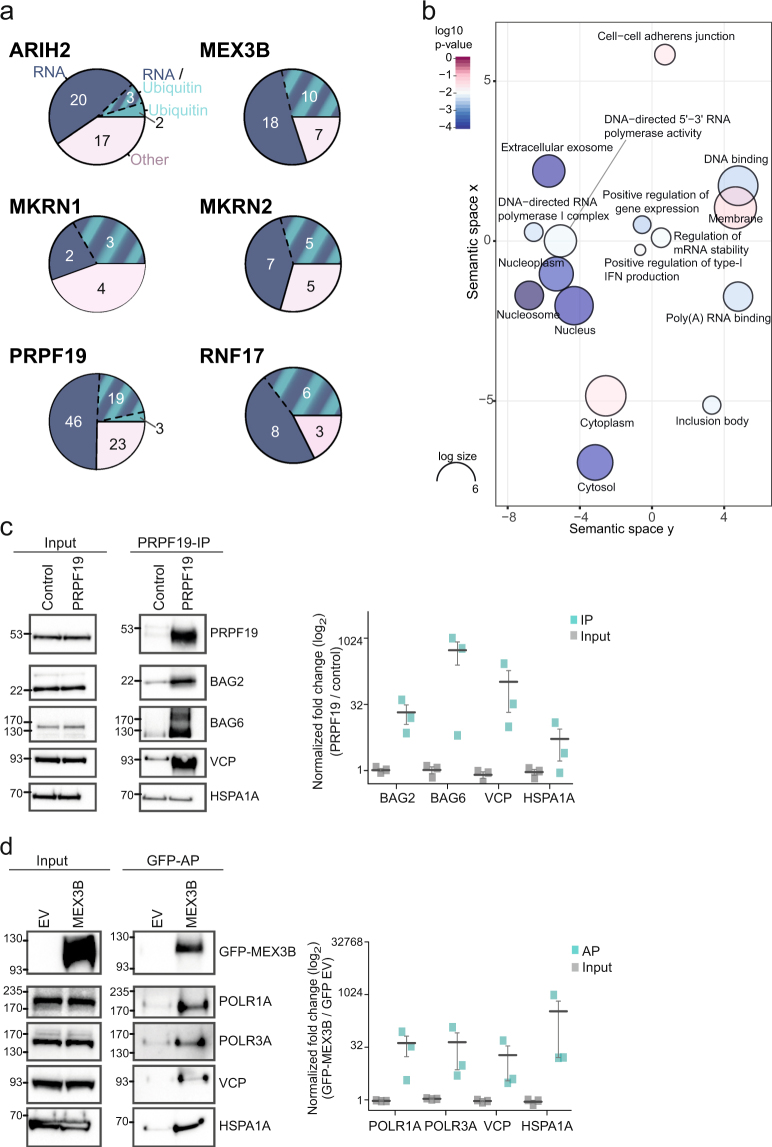
The RBULs link posttranscriptional processes to the ubiquitin system. (**a**) The BP and MF GO terms of the interactors of the six RBULs were grouped into the categories “RNA” (blue), “Ubiquitin” (turquoise), and “Other” (rose). The distribution of the categories among the interactomes is shown. (**b**) A summary of GO terms for MEX3B interaction partners is shown. (**c**,**d**) Validation of RBUL interaction partners by pulldowns and Western blot. (**c**) Endogenous PRPF19 was pulled down with a PRPF19-specific antibody from HEK293T cells. Experiments omitting the antibody served as control. Western blot analysis was performed with antibodies specific against BAG2, BAG6, VCP, and HSPA1A, as well as against PRPF19 itself to validate the immunoprecipitation (IP). Left: Cropped images of input and IP samples (replicate 1). Images of full membranes and different exposure times for all antibodies and replicates are presented in Supplementary Figure 4a–c. Right: Quantifications of the PRPF19-specific IPs normalized to control of three independent biological replicates are shown in a dot plot, including mean and standard error (s.e.m.). (**d**) GFP (empty vector, EV) and GFP-MEX3B were expressed in HEK293T cells and pulled down with a GFP-specific antibody. Western blot analysis was performed using specific antibodies against POLR1A, POLR3A, VCP, and HSPA1A, as well as GFP. Left: Cropped images of input and AP samples (replicate 1). Images of full membranes and different exposure times for all antibodies are presented in Supplementary Figure 4d–f. Right: Quantification of the APs normalized to EV are shown as in (**c**).

Using our adapted AP, we identified between 9 and 42 stable interaction partners which displayed a z-score ≥ 2 with the different RBULs (ARIH2, 42; MEX3B, 35; MKRN1, 9; MKRN2, 17; RNF17, 17; Figs 2f and 3, Suppl. Tables 1, 2, 3). Among these, we find a small number of likely contaminants, including HSP70 chaperone family members, such as HSPA1A, that act as disaggregases for newly translated proteins and thus represent common contaminants in AP experiments^29,30^. In line with the notion that these interactions could be a secondary effect of ectopic RBUL expression, HSPA1A reproducibly interacted with GFP-tagged PRPF19 and MEX3B, but showed only minor interaction with endogenous PRPF19 in independent validation experiments (Fig. 4c,d, Suppl. Figure 3b).

In order to assess the functional coherence of the identified interactomes, we employed the GO similarity scoring approach. This confirmed that the interactomes of the six RBULs are enriched for RBUL-interactor pairs with WI ≥ 0.414, suggesting that they generally represent physiological interactors. The exception is RNF17, which is mainly annotated with superordinate GO terms such as ‘cellular process’ and hence not appropriate for GO similarity scoring. The applied cut-off (z-score ≥ 2) consistently falls within the margin of specific enrichment, supporting the choice of this threshold to detect meaningful interactions. For comparison, we subjected the five RBULs to the conventional SILAC-AP with and without RNase treatment (Suppl. Figure 2a,b, Suppl. Tables 1 and 3). Similar to the initial observation with PRPF19, our adapted AP consistently outperforms the conventional SILAC-AP in the Precision-Recall evaluation for these RBULs (Fig. 2f). Accordingly, we find hardly any overlap between the interactome lists from the two approaches (Suppl. Figure 2c). This nicely illustrates the conceptual differences of the two protocols and their target interactions, but may also result at least in parts from the inherent limitations of quantitative proteomics in terms of coverage and sensitivity^31^.

Integration of all datasets revealed that a major fraction of the interactors of each RBUL are unique and not shared with any other tested RBUL (38% [RNF17] - 88% [ARIH2]; Fig. 3), suggesting broad functional divergence. In order to get insights into their cellular functions, we used GO annotations (Biological Process and Molecular Function) to classify the interaction partners into the categories ‘RNA’, ‘Ubiquitin’ and ‘Other’. Notably, the interaction partners of all studied RBULs are implicated into both, posttranscriptional processes as well as the ubiquitin system (Fig. 4a). In accordance with their ubiquitin ligase function, the interaction partners of RBULs are linked to core functionalities of the ubiquitin system. First, we find ubiquitin itself (RPS27A) as a stable interaction partner of all six RBULs, which could be indicative of auto-ubiquitylation. In addition, cross-targeting between different E3 ligases has also been described as a mode of activity regulation^32^. In line with this notion, we observe stable associations with other E3 ligases, including RNF20, CUL5, and MID1 (ARIH2) as well as HUWE1 (MEX3B and PRPF19). For PRPF19, we also identify an interaction with the ubiquitin-specific peptidase USP19 (Fig. 3). Moreover, we detect the ubiquitin-dependent co-chaperone VCP (also known as p97) in stable association with all RBULs (Fig. 3). VCP is an ATPase that structurally remodels ubiquitylated proteins before their degradation through the ubiquitin-proteasome or the autophagy-lysosome pathway^33^. We verified the interaction of VCP with GFP-tagged MEX3B and PRPF19 as well as endogenous PRPF19 using pulldown experiments followed by Western blot (Fig. 4c,d, Suppl. Figure 3b).

In addition to the ubiquitin-related functions, mapping of the RBUL interactomes reveals links to different posttranscriptional processes. For instance, several RBULs interact with translation initiation factors, including EIF4G1 (PRPF19 and MEX3B) and EIF1AX (ARIH2), and ribosomal proteins, such as RPLP2 (RNF17), RPS27L (MKRN1), RPL37A, and RPS12 (MKRN2). More generally, comparison of the RBUL interaction profiles with several large-scale mRNA interaction profiling screens^1–3^ reveals that 68 out of the total of 170 interactors (40%) have been found in direct contact with poly(A) + RNA, underlining the strong commitment of the studied RBULs to RNA-mediated processes.

Interestingly, a fraction of the identified interactors is involved in other cellular processes, such as protein transport, cell cycle or DNA damage repair (‘Other’, Fig. 4a). Additionally, stable interaction of PRPF19 and other RBULs with RNA polymerase components predicts a function in transcriptional regulation (POLR2A, POLR2B [PRPF19], POLR1A, POLR1C, POLR2H [MEX3B], POLR3A [PRPF19, MEX3B and ARIH2]; Fig. 3). As PRPF19 has been previously implicated in the regulation of transcription^34,35^, this observation underlines the capacity of our approach to identify physiologically functional networks. In line with a putative role in transcriptional regulation, we also identify multiple components of the prefoldin-like complex as stable PRPF19 interactors (PFDN1, PFDN2, VBP1/PFDN3, PFDN4 and PFDN6). Prefoldin acts as a co-chaperone in the cytoplasmic assembly of cytoskeletal and non-cytoskeletal complexes, but also modulates transcriptional activation when present in the nucleus^36^. Moreover, we find multiple proteins that are involved in the DNA damage response to interact with PRPF19, such as DDB1, MDC1, CDC5L, SNW1 and DNAJA1. In line with this notion, PRPF19 has been recently implicated in the DNA damage response by ubiquitylation of the single-strand DNA-binding protein RPA^37^. Finally, we also detect a stable interaction of PRPF19 with all subunits of the BAT3 complex, composed of BAG6/BAT3, UBL4A and GET4/TRC35, which is involved in chaperoning and degrading ER-associated proteins^38^. To substantiate this finding, we validated the interaction of BAG6 in pulldown experiments against endogenous as well as GFP-tagged PRPF19 followed by Western blot (Fig. 4c, Suppl. Figure 3b). Similarly, we also confirmed the interaction with BAG2 which was previously implicated to function together with VCP in proteolytic pathways^33,39^.

Taking MEX3B as an example, we used the DAVID database to assess coherent functions of its interactors^40^. Consistent with previous reports^41,42^, we find that the interactors of MEX3B are involved in biological processes such as ‘regulation of mRNA stability’ (adjusted *p*-value < 0.05, Benjamini-Hochberg correction) (Figs 3 and 4b, Suppl. Table 4). Among the GO Molecular Function (MF) terms, ‘poly(A) RNA binding’ appears as a predominant term, reflecting the stable association of MEX3B with proteins involved in RNA metabolism, such as DHX36 and TNRC6A. Finally, a significant fraction of the identified interactors suggest a direct role of MEX3B in the regulation of transcription, including subunits of RNA polymerases (pol) I, II and III, regulatory proteins such as the transcriptional repressor ZGPAT, as well as multiple histone-associated proteins. We validated the interactions with two RNA pol I and III subunits for GFP-MEX3B (Fig. 4d). Altogether, these findings place MEX3B and the other RBULs into the context of several pathways of transcriptional and posttranscriptional gene regulation, forming a basis for future studies regarding the role of RBULs in these cellular processes. In summary, we were able to identify novel protein-protein interaction partners of six human RBULs and link them to cellular pathways by applying our adapted AP protocol followed by bioinformatics analyses.

## Discussion

In this study, we analyzed the interaction profiles of six human RBULs containing different RNA-binding domains using a modified AP approach coupled to LC-MS/MS. Previous studies have suggested that RBPs are particularly prone to engage in non-physiological interactions that hamper the identification of physiological interaction partners^7,8^. For instance, the reassembly of ribonucleoprotein complexes in cell lysates can lead to false positive results when studying the *in vivo* interaction partners of RBPs^7,43^. Moreover, the disruption of cellular compartmentalization during cell lysis promotes artificial associations that would be impossible *in vivo* due to spatial separation^44^. The propensity of RBPs for re-assortment is augmented by the prevalence of disordered regions in these proteins, which often contain repetitive sequences and low-complexity domains. Such disordered regions can facilitate RNA recognition but also promote association with other molecules^3,6^. In line with this notion, it has been found that many RBPs undergo a concentration-dependent phase transition into a hydrogel-like state^8,45,46^. In the cellular context, this might play an important role in driving the assembly of non-membrane-bounded subcellular structures, such as RNA granules or liquid-like clusters. However, this biophysical behavior is likely to interfere with reliable proteomic characterizations, as the granules reversibly aggregate and disaggregate in solution which may entrap proteins in non-physiological associations^46,47^. Altogether, the biochemical and biophysical properties of RBPs make them challenging targets for unbiased protein-protein interaction profiling.

In order to overcome these obstacles, we modified the conventional SILAC-based AP workflow to make it more suitable for RBPs. By combining the differentially SILAC-labeled cell populations prior to cell lysis and AP, the SILAC ratios become indicative of the stability of the detected interaction. Only kinetically stable interactions will maintain high SILAC ratios, while any association that newly forms or changes in the lysate will display an equalized/background SILAC ratio. Our approach thereby advances the mixing of samples to an earlier step compared to PAM-SILAC, which is commonly employed to study stable protein interactions^48^. Previous studies established that a mixing of samples at different stages of the AP protocol allows to reliably distinguish between stable and transient interactions, a strategy that was successfully applied e.g. to dissect the dynamic interactome of transcription factor complexes^49^ or the human 26 S proteasome^9^.

In order to facilitate quick, clean and reproducible immunoprecipitations, our adapted AP approach relies on the ectopic expression of GFP-tagged bait proteins. GFP has been evaluated as a suitable tag of choice for quantitative proteomics due to its minimal non-specific binding to cellular proteins^30^. Moreover, it can be combined with control cells expressing unfused GFP, which has been suggested as an important internal control to further eliminate non-specific contaminants^50^. Our Western blot experiments show that the GFP-tagged RBULs display a certain deviation in expression levels compared to the endogenous counterpart (37-fold less and 13-fold more for PRPF19 and MEX3B, respectively; Suppl. Figure 3a), which might partially impair protein folding and function. Accordingly, we detect recurrent interactions with several HSP70 family members, which can function as disaggregases for newly translated proteins and were previously reported as recurrent GFP-associated contaminants^29,30^. Nevertheless, we reliably recover a large number of known physiological interaction partners, indicating that the overall functionality of the GFP-tagged RBULs is preserved. Moreover, the GFP-tagged RBULs mostly localize to the correct cellular compartment, supporting the notion that the majority of the tagged proteins is properly folded and delivered. It is important to note that our protocol is also compatible with standard antibodies against endogenous proteins, which usually support an efficient immunoprecipitation on a similar timescale. This would allow to circumvent the ectopic expression of tagged bait proteins in future applications.

In order to validate the performance of our approach, we used the well-characterized RBUL PRPF19 as an example. PRPF19 has been described to engage in at least three different sub-complexes that confer distinct cellular functions in human cells^51^. Using the adapted AP, we recover the interactions of PRPF19 with the NTC core complex as well as components of the XAB2 complex, a second PRPF19 sub-complex involved in genome maintenance^34^. Moreover, we identify multiple components of the ubiquitin-proteasome system such as VCP, HSP90AB1, UBL4A, GET4 as well as several proteasome subunits (PSMC1, PSMC2, PSMC5, PSMC6, PSMD2). Although we do not detect PSMB4, a subunit of the catalytic core of the proteasome that was previous reported to interact with PRPF19, the identified interaction partners strongly support the idea of a proteasome-dependent function of PRPF19-mediated ubiquitylation. The interactions with proteasome components were previously interpreted as a potential link of PRPF19 to protein degradation via substrate delivery to the proteasome^51^. Finally, the observation that PRPF19 interacts with the BAT3 complex, which we independently validated for both the GFP-tagged as well as the endogenous protein, suggests a role in chaperoning and targeting proteins within the cytosol^38^. In summary, the recovery of known PRPF19 interactors supports the validity of our approach to identify physiological protein interactions from the native environment of the cell.

The identified protein-protein interactions could be regulators as well as ubiquitylation substrates of the RBUL. For instance, PRPF19 was previously shown to transfer a non-proteolytic K63-linked ubiquitin chain to PRPF3 within the U4 snRNP, thereby stabilizing the U4/U6.U5 tri-snRNP during spliceosome remodeling^51,52^. Subsequent de-ubiquitylation is a prerequisite for spliceosome activation. In the adapted AP, we do not identify PRPF3; however, PRPF8 as well as several other components of the tri-snRNP are found (such as SNRPE, SNRPD3, SNRNPD40 and SNRNP200), which might represent regulators or substrates of PRPF19. To distinguish ubiquitylation substrates from regulators of protein function, further experiments including ubiquitin remnant profiling in ubiquitin ligase knockdown cells or pulldowns using catalytically dead mutants could be performed^53,54^.

Using the adapted AP, we characterized the core interactomes of five additional RBULs, including ARIH2/TRIAD1, MEX3B, MKRN1, MKRN2 and RNF17. In total, we detected 170 interactors, which will be a valuable resource for future studies on RBUL functions in human cells. In line with the composite domain architecture, our results suggest that all tested RBULs interact both with components of the ubiquitin system as well as with RBPs involved in different posttranscriptional processes (116 out of 170 interactors belonging to either category; 68%). For example, the hnRNP family of RNA-binding proteins has been reported to interact with ubiquitin ligases such as TRIM28, PRPF19, and CUL1^55^. In accordance, we find PRPF19 in stable association with HNRNPC.

There are multiple known examples of regulatory associations between ubiquitylation and RNA. On the one hand, ubiquitylation can control the activity of large RNA processing machineries, as mentioned above for the modification of PRPF3 by PRPF19 during spliceosome remodeling. Similarly, non-proteolytic ubiquitylation of CNOT7 by the RBUL MEX3C activates de-adenylation by the CCR4-NOT machinery, which is the rate-limiting step in eukaryotic mRNA decay. Notably, the ubiquitin modification has no effect on basal de-adenylation activity of CNOT7 but is important for the decay of specific mRNAs that are recognized by MEX3C on the level of RNA binding^55^. The RNA-binding/ubiquitylation-mediated CCR4-NOT activation thus introduces an additional layer of specificity for distinct RNAs that might be relevant under certain cellular conditions such as during cell differentiation or disease^56^. On the other hand, a reverse relationship has also been observed in which the RNA functions as a regulatory molecule to control activities within the ubiquitin system. For instance, RNA binding of the Roquin paralog RC3H2 influences its E3 ubiquitin ligase function *in vitro* ^57^. Moreover, the long non-coding RNA *HOTAIR* was found to act as a molecular assembly platform for protein ubiquitylation^58^. This is achieved through adjacent RNA binding of the RBULs DZIP3 and MEX3B together with their respective client proteins Ataxin-1 and Snuportin-1, which facilitates their ubiquitylation and accelerates their degradation, e.g. during cellular senescence. It is conceivable that similar mechanisms are at work to control transcription and translation.

Notably, we find that RBULs form stable interactions with RNA polymerase subunits as well as with components of the translation machinery. In line with the notion that the RBULs could be involved in translational regulation, several studies demonstrated a role for ubiquitin as a modulator of translation. For instance, K63-linked poly-ubiquitylation of ribosomal proteins and translation elongation factors was recently shown to promote translation during oxidative stress in baker’s yeast^59^. Similarly, 40S ribosomal proteins in human cells were found to be ubiquitylated due to the activation of the unfolded protein response and inhibition of translation^60^. Moreover, the E3 ubiquitin ligase CUL3 has been implicated in the ubiquitin-dependent formation of a ribosome modification platform that alters the translation of specific mRNAs^61^. Future studies will be needed to understand the molecular mechanisms by which RBULs link ubiquitylation to posttranscriptional processes. Our results provide evidence for an extensive crosstalk between these two modes of gene regulation.

In summary, we conclude that the adapted AP approach is capable of detecting so far unknown protein-protein interactions of RBPs and enables embedding those protein-protein interactions into a functional context. It proves particularly valuable for studying RBPs that are prone to engage into non-physiological interactions during cell lysis. Our approach thereby adds a useful tool to complement existing protocols.

## Material and Methods

### Cell culture

HEK293T cells were purchased from DSMZ (Catalog no. ACC 635) and maintained in DMEM (Life Technologies, 21969035) complemented with 1% penicillin/streptomycin (Life Technologies, 15140-122), 1% L-glutamine (Life Technologies, 25030-029) and 10% fetal bovine serum (Life Technologies, A15-101). All cells were cultured at 37 °C in a humidified incubator containing 5% CO_2_. For SILAC labeling, cells were cultured in media containing either L-arginine and L-lysine (light SILAC label), L-arginine (^13^C_6_) and L-lysine (^2^H_4_) (medium SILAC label), or L-arginine (^13^C_6_-^15^N_4_) and L-lysine (^13^C_6_-^15^N_2_) (heavy SILAC label) (Cambridge Isotope Laboratories).

### Vectors/plasmids

The following entry vectors, suitable for Gateway Cloning, were obtained from the IMB Core Facility ORFeome Collection^62^: pENTR221-ARIH2, pENTR201-MEX3B, pENTR221-MKRN1, pENTR221-MKRN2, pENTR221-PRPF19 and pENTR223.1-RNF17. Coding sequences of six RNA-binding ubiquitin ligases were cloned into the mammalian expression vector pMX-DEST53-IP-GFP by LR Gateway cloning according to manufacturer’s recommendations (Gateway® LR Clonase® II Enzyme mix; Life Technologies, 11791-100), then ectopically expressed in HEK293T cells using Polyethylenimine MAX 4000 (Polysciences, 24885-2).

### Immunoprecipitations

For GFP-APs, HEK293T cells transiently expressing GFP empty vector or a GFP-tagged RBUL were used. For endogenous APs, untransfected HEK293T cells were used. The cells were washed with ice-cold phosphate-buffered saline (PBS) and lysed in modified RIPA (mRIPA) buffer (50 mM Tris-HCl pH 7.5, 150 mM NaCl, 1 mM EDTA, 1% NP-40, 0.1% sodium deoxycholate). The mRIPA buffer was supplemented with protease inhibitors (protease inhibitor cocktail, Sigma). Lysates were cleared at 16,000 × g for 15 min. Protein concentrations of the cleared lysates were estimated by BCA Protein Assay (Thermo Scientific). GFP-trap agarose beads (Chromotek) were washed in mRIPA buffer before they were incubated with the cleared lysate for 1 h at 4 °C. After three washes with NET buffer (50 mM Tris-HCl pH 7.5, 150 mM NaCl, 5 mM EDTA, 0.1% Triton X-100) and three washes with ultrapure water, the beads were resuspended in LDS sample buffer (Life Technologies) and boiled at 70 °C for 10 min.

### Western blotting

Denatured proteins were separated by SDS-PAGE on a NuPAGE 4–12% Bis-Tris protein gel (Life Technologies) and transferred to a 0.45 µm nitrocellulose membrane (VWR). For detection, HRP-conjugated secondary antibodies and WesternBright Chemiluminescent Substrate (Biozym Scientific) or SuperSignal West Pico Chemiluminescent Substrate (Life Technologies) were used. Western blots were quantified by determining the background-subtracted densities of the protein of interest using Image J^63^. The signal from the IP (against endogenous PRPF19) or AP (against GFP-tagged PRPF19 and MEX3B) was normalized to the respective control samples omitting the antibody or expressing the empty vector, respectively.

### Antibodies

The following antibodies were used: anti-GFP (B-2 clone; Santa Cruz; sc-9996), anti-HSP70 (Enzo Life Sciences; N15F2-5), anti-BAG6 (Cell Signaling Technology, 8523), anti-VCP (Cell Signaling Technology; 2649), anti-BAG2 (Sigma Alrich; HPA018862), anti-PRPF19 (Abcam; ab27692), anti-POLR1A (Santa Cruz; sc-48385), anti-POLR3A (D5Y2D; Cell Signaling Technology, 12825).

### Immunofluorescence microscopy

HEK293T cells were seeded on microscopy cover slips and transfected with GFP-tagged RBULs using Polyethylenimine “Max” (Mw 4,000) (Polysciences Inc). Cells were washed with ice-cold PBS and fixed with 4% paraformaldehyde (Affymetrix) for 20 min. Cells were stained with DAPI (Sigma) and rinsed in PBS, wash buffer (10 mM Tris-HCl, pH 7.5) and water. The samples were mounted with ProLong Diamond Antifade Mountant (Life Technologies). For analysis, a Leica SP5 II confocal system (Leica Microsystems GmbH) with a 63× oil immersion NA1.4 objective lens was used, and four pictures were taken per frame for each RBUL. Images were processed in Fiji^64^.

### Sample preparation for the conventional SILAC workflow

HEK293T cells transiently expressing GFP were cultured in light SILAC medium, while cells expressing GFP-tagged RBULs were cultured in medium and heavy SILAC medium. In the conventional SILAC-AP experiments, enriched protein fractions extracted from GFP-RBUL-expressing heavy isotope-labeled cells were used for RNase digestion. The cells were washed with ice-cold PBS and lysed in mRIPA buffer. The mRIPA buffer was supplemented with protease inhibitors, 10 mM N-ethylmaleimide (NEM), 1 mM sodium orthovanadate, 5 mM β-glycerophosphate, and 5 mM sodium fluoride. Lysates were cleared at 16,000 × g for 15 min. The Pierce BCA Protein Assay Kit (Thermo Fisher) was used to estimate protein concentrations of the cleared lysates. GFP-trap agarose beads were washed in mRIPA buffer before they were incubated with the cleared lysate for 1 h at 4 °C. After two washes in NET buffer, the heavy SILAC labeled lysate was incubated with 0.5 U/µl RNase A (Qiagen) and 20 U/µl RNase T1 (Thermo Fisher Scientific) for 30 min at 4 °C, while the light and medium SILAC-labeled protein precipitates were kept on ice. All three differentially SILAC-labeled pulldown samples were washed twice with NET buffer and twice with ultrapure water, then combined, and washed once more with ultrapure water. The beads were resuspended in digestion buffer and incubated with 10 mM dithiothreitol (DTT; Sigma, D5545) for 20 min at RT and with 50 mM 2-Chloroacetamide (CAA; Sigma, C0267) for 20 min at RT in the dark^20^.

### Sample preparation for the adapted SILAC-based AP protocol

HEK293T cells transiently expressing GFP were cultured in light SILAC medium, while cells expressing GFP-tagged RBULs were cultured in heavy SILAC medium. Cells were washed with ice-cold phosphate-buffered saline. Light and heavy SILAC labeled cells were mixed in equal amounts. Then, the combined cell fraction was lysed in mRIPA (as described above). Lysates were cleared at 16,000 × g for 15 min. Pierce BCA Protein Assay Kit was used to estimate protein concentrations of the cleared lysates. GFP-trap agarose beads were washed in mRIPA buffer before they were incubated with the cleared lysate for 1 h at 4 °C. After three washes with NET buffer and three washes with ultrapure water, the beads were resuspended in digestion buffer and incubated with 10 mM DTT for 20 min at room temperature (RT) and with 50 mM CAA for 20 min at RT in the dark^20^.

### MS sample preparation

The enriched proteins were digested on-bead with trypsin for 2 h at RT. Supernatants were transferred to new microcentrifuge tubes, while the beads were re-incubated with digestion buffer for 5 min at RT. Afterwards, supernatants were combined with the supernatants collected before and trypsin was added to continue protein digestion overnight at RT^20^. For two PRPF19 experiments (replicates 1 and 2 of the conventional SILAC-AP and the adapted AP), proteins were resolved by SDS-PAGE on a NuPAGE 4–12% Bis-Tris protein gel (Thermo Fisher Scientific) and submitted to in-gel digest using trypsin. Subsequently, peptides were extracted from the gel. To concentrate, clear and acidify the peptides, they were bound to C18 StageTips as described previously^65,66^.

### MS analysis

Peptide fractions were analyzed on a quadrupole Orbitrap mass spectrometer (Thermo Q Exactive Plus, Thermo Scientific) coupled to an UHPLC system (EASY-nLC 1000, Thermo Scientific)^67^. Peptide samples were separated on a C18 reversed phase column (length: 20 cm, inner diameter: 75 µm, bead size: 1.9 µm) and eluted in a linear gradient from 8 to 40% acetonitrile containing 0.1% formic acid in 105 min. The mass spectrometer was operated in data-dependent positive mode, automatically switching between MS and MS^2^ acquisition. In the Orbitrap, the full scan MS spectra (m/z 300–1650) were acquired. Sequential isolation and fragmentation of the ten most abundant ions was performed by higher-energy collisional dissociation (HCD)^68^. Peptides with unassigned charge states, as well as with charge states less than +2 were excluded from fragmentation. The Orbitrap mass analyzer was used for acquisition of fragment spectra.

### Peptide identification and quantification

Using the MaxQuant software (version 1.5.28), raw data files for each RBUL (conventional and adapted AP) were analyzed and peptides were identified^24^. Parent ion and MS^2^ spectra were compared to a database containing 92,578 human protein sequences obtained from UniProtKB, released in May 2016, coupled to the Andromeda search engine^69^. Cysteine carbamidomethylation, oxidation, and NEM were set as fixed modifications. The mass tolerance for the spectra search was set to be lower than 6 ppm in MS and 20 ppm in HCD MS^2^ mode. Spectra were also searched with strict trypsin specificity and allowing up to three miscleavages. Site localization probabilities were determined by MaxQuant using the PTM scoring algorithm as described previously^21,69^. Filtering of the dataset was based on the posterior error probability to arrive at a false discovery rate below 1% estimated using a target-decoy approach^70^. Proteins that were categorized as “only identified by site”, known contaminants and reverse hits were removed. Only proteins identified with at least two peptides (including at least one unique peptide) and a SILAC ratio count at least two were used for analysis. The SILAC ratios were log_2_ transformed and converted into an asymmetric z-score based on the mean and interquartile range of the distribution as described previously^21^. In order to extract interactors for the six RBULs, we log_2_ transformed the SILAC ratios. For proteins that were detected in at least two replicate experiments for a given RBUL and AP protocol, we applied a cut-off at mean z-score ≥ 2 to identify putative interaction partners^21,22^. Interactions from the conventional SILAC-AP experiments were additionally classified as RNA-dependent or RNA-independent based on z-score ≤ −1 or >−1, respectively, when comparing GFP-RBUL + RNase vs. GFP-RBUL SILAC ratios determined by conventional SILAC-AP. Ratio-Intensity plots were created in R (version 3.2.3).

### GO similarity approach and GO enrichment analysis

The guilt-by-association principle states that proteins that interact with each other usually participate in the same biological process or pathway^24^. Under this premise, we considered the interactions between RBULs and putative protein partners as biologically meaningful if the Gene Ontology (GO) similarity between the putative interactors was high. We used the R package GOSemSim^25^ to compute GO similarities between RBULs and their interaction partners based on the Wang’s Index (WI) comparing their Biological Process (BP) GO terms (state June 2017). WI was developed specifically for GO and emits similarities that are consistent with manually curated GO-gene associations^26^. To benchmark our approach, we calculated the WI distribution of interacting proteins in INstruct, a high-quality database of structurally resolved protein interactions, containing 3,354 proteins (N) with 6,093 edges (L, interactions) between them^27,28^. As a negative control, we used all disconnected protein pairs that are not connected by edges within the INstruct network (N * (N − 1)/2-L = 5,616,888 non-edges). As expected, the BP GO similarity of connected protein pairs in the INstruct network is significantly higher than that of disconnected pairs (p-value < 2.2 × 10^−16^, Mann-Whitney U test). Based on the control distribution, we estimated an empirical false discovery rate (FDR) and a cut-off at FDR <10%, corresponding to WI = 0.414 (Fig. 2a).

In order to construct the Precision-Recall curves, we sorted the list of RBUL-interactor pairs in decreasing order by their normalized SILAC ratios from a given AP approach, and considered an interaction as ‘*true*’ i.e. functionally coherent if its WI was at or above 0.414. We then progressively went over the entire range of ratios, assessing an increasing number of protein interaction pairs (using stepwise increase of one interaction pair in each iteration). In each iteration, the fraction of *true* protein pairs from the total determines the ‘Precision’, while the fraction of *true* protein pairs at the current step from the total of *true* interactions in the list determines the ‘Recall’. After the evaluation of the full range of SILAC ratios, all Precision-Recall value pairs were used to construct a curve that measures the ability of the AP approach to preferentially assign high SILAC ratios to functionally coherent interactions. If no GO term was available for a prey, the WI was set to −1.

All computational analyses were executed on a Lenovo ThinkPad 64-bit with 7.7 GB of RAM and an Intel Core i7-4600U CPU @ 2.10 GHz × 4, running Ubuntu 16.04 LTS.

### Functional annotation of RBUL interactors

To assess the involvement of interacting proteins in posttranscriptional (‘RNA’) or in ubiquitin-mediated processes (‘Ubiquitin’, Fig. 4a) we used a manually curated list of GO terms for ‘Biological Process’, ‘Molecular Function’ and ‘Cellular Component’. GO enrichment analyses were performed using the DAVID (Database for Annotation, Visualization and Integrated Discovery) tool for all three GO domains^40^. Enriched GO terms (adjusted *p-*value < 0.05, Benjamini-Hochberg correction) were visualized using REVIGO^71^ (Reduce & Visualize Gene Ontology), allowing medium GO term similarity (Fig. 4b). All identified RBUL-interactor pairs with a z-score ≥ 2 from the adapted AP (170 in total) were queried against the Human Integrated Protein-Protein Interaction Reference (HIPPIE) database^23^. Out of 170 interactions in total, only 19 were reported to date. HIPPIE confidence scores, number of interactions reported for each of the six RBULs in HIPPIE and the kind of experiment used to measure in combination with PMIDs and reporting databases were extracted from HIPPIE.

### Data availability

All datasets generated in the current study are available from the corresponding author on request.

## Electronic supplementary material

Supplementary Material

Supplementary Table 1

Supplementary Table 2
