# Supplementary material for: Interaction profiling of RNA-binding ubiquitin ligases reveals a link between posttranscriptional regulation and the ubiquitin system
Source: Sci Rep. 2017 Nov 29;7:16582. doi: 10.1038/s41598-017-16695-6 (PMC5707401; doi:10.1038/s41598-017-16695-6)
Supplement: Supplementary file 1 — Supplementary Material [file 41598_2017_16695_MOESM1_ESM.pdf]

Interaction profiling of RNA-binding ubiquitin ligases reveals a link between posttranscriptional regulation and the ubiquitin system

SUPPLEMENTARY MATERIAL

Andrea Hildebrandt[1], Gregorio Alanis-Lobato[1,2], Andrea Voigt[1], Kathi Zarnack[3], Miguel A. Andrade-Navarro[1,2], Petra Beli[1*] and Julian König[1*]

[1]Institute of Molecular Biology (IMB), Ackermannweg 4, 55128 Mainz, Germany

[2]Faculty of Biology, Johannes Gutenberg University, Gresemundweg 2, 55128 Mainz, Germany

[3]Buchmann Institute for Molecular Life Sciences (BMLS), Goethe University Frankfurt, Max-von-Laue-Str. 15, 60438 Frankfurt, Germany

*Correspondence should be addressed to:

Petra Beli: p.beli@imb-mainz.de

Julian König: j.koenig@imb-mainz.de

Content:

Supplementary Figures 1 – 4 (including legends)

Legends for Supplementary Tables 1 – 2

Supplementary Tables 3 – 6 (including legends)

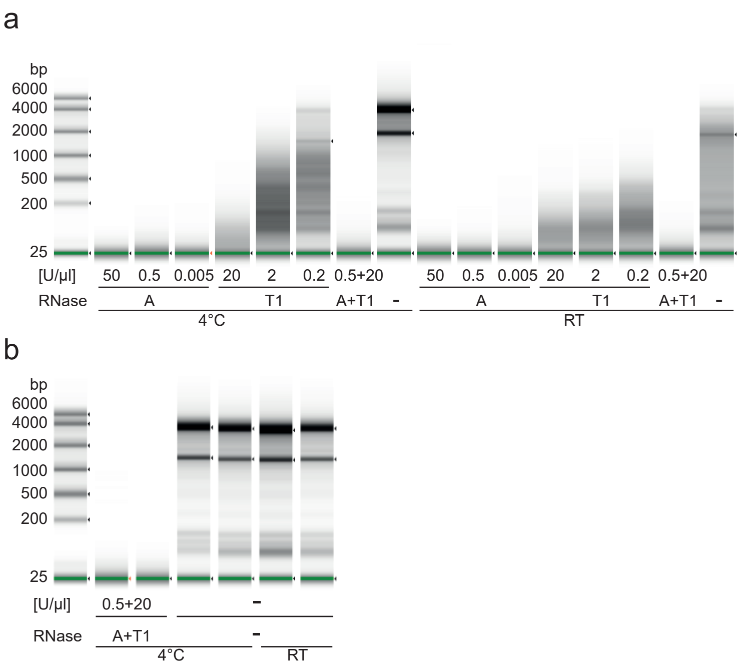

Supplementary Figure 1. RNase optimization for the conventional SILAC-AP workflow. Cells were lysed and incubated with different concentrations of RNase A and/or RNase T1 for 30 min at 4°C or room temperature (RT). RNA was isolated using Trizol and analyzed by capillary gel electrophoresis. **a.** The level of RNA degradation using different RNase concentrations and combinations at 4°C or RT was tested. **b.** RNA treated with conditions chosen from A (0.5 U/µl RNase A + 2 U/µl RNase T1, 4°C, 30 min) was compared to undigested RNA levels kept at 4°C or RT for 30 min in duplicates by capillary gel electrophoresis.

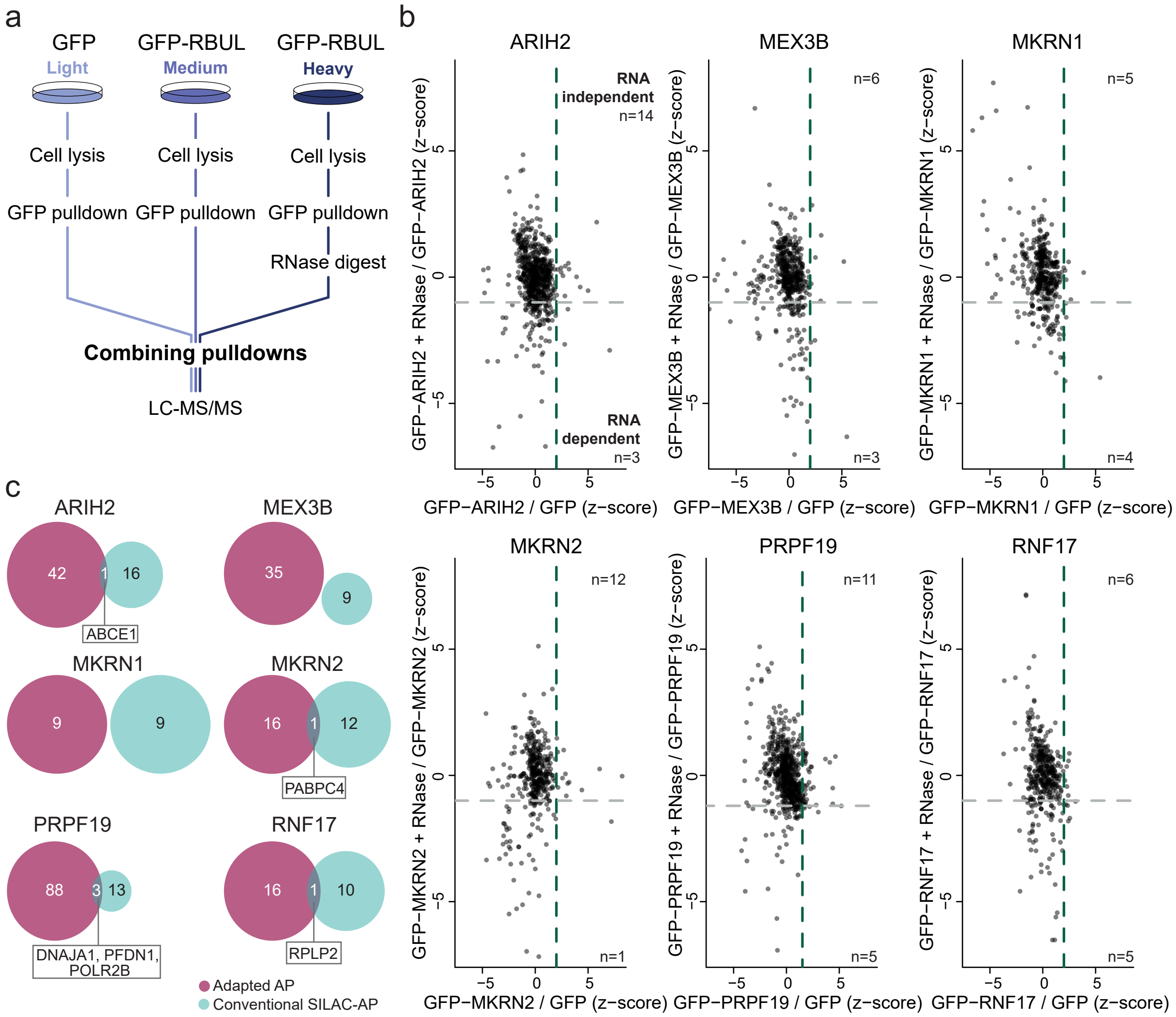

Supplementary Figure 2. Conventional SILAC-AP in combination with RNase digest enables differentiation between RNA-dependent and RNA-independent protein interactions of the six studied RBULs. a. In the conventional SILAC-AP in combination with RNase digest, GFP alone was expressed in light-isotope labeled cells (light blue), while a GFP-tagged RBUL was expressed in medium-isotope (medium blue) and heavy-isotope (dark blue) labeled cells. Lysates were subjected to GFP-specific AP. After washing, heavy-isotope labeled enriched proteins bound to the GFP-trap agarose beads were incubated with RNase A and RNase T1 for 30 min at 4°C. Light- and medium-isotope labeled proteins were kept on ice without RNase digest. After washing, enriched proteins were mixed in a 1:1 ratio, digested on-bead with trypsin, and analyzed by LC-MS/MS. **b.** Conventional SILAC-APs are shown in dependence on RNase digest. GFP-RBUL vs. GFP SILAC ratios after z-score normalization are plotted against z-score normalized SILAC ratios of GFP-RBUL with RNase digest vs. GFP-RBUL. The dashed lines indicate the applied cut-offs at z-score ≥ 2 for GFP-RBUL over GFP (green; x-axis) and at z-score ≤ -1 for GFP-RBUL + RNase over GFP-RBUL (grey; y-axis). n, number of interactors in the two categories. **c.** Comparisons of interaction partners for the adapted AP and the conventional SILAC-AP are shown. Proteins are only considered for this analysis if they are detected in at least two replicates and pass the z-score ≥ 2 cut-off for both AP approaches. Proteins detected in both AP approaches are named below the Venn diagrams.

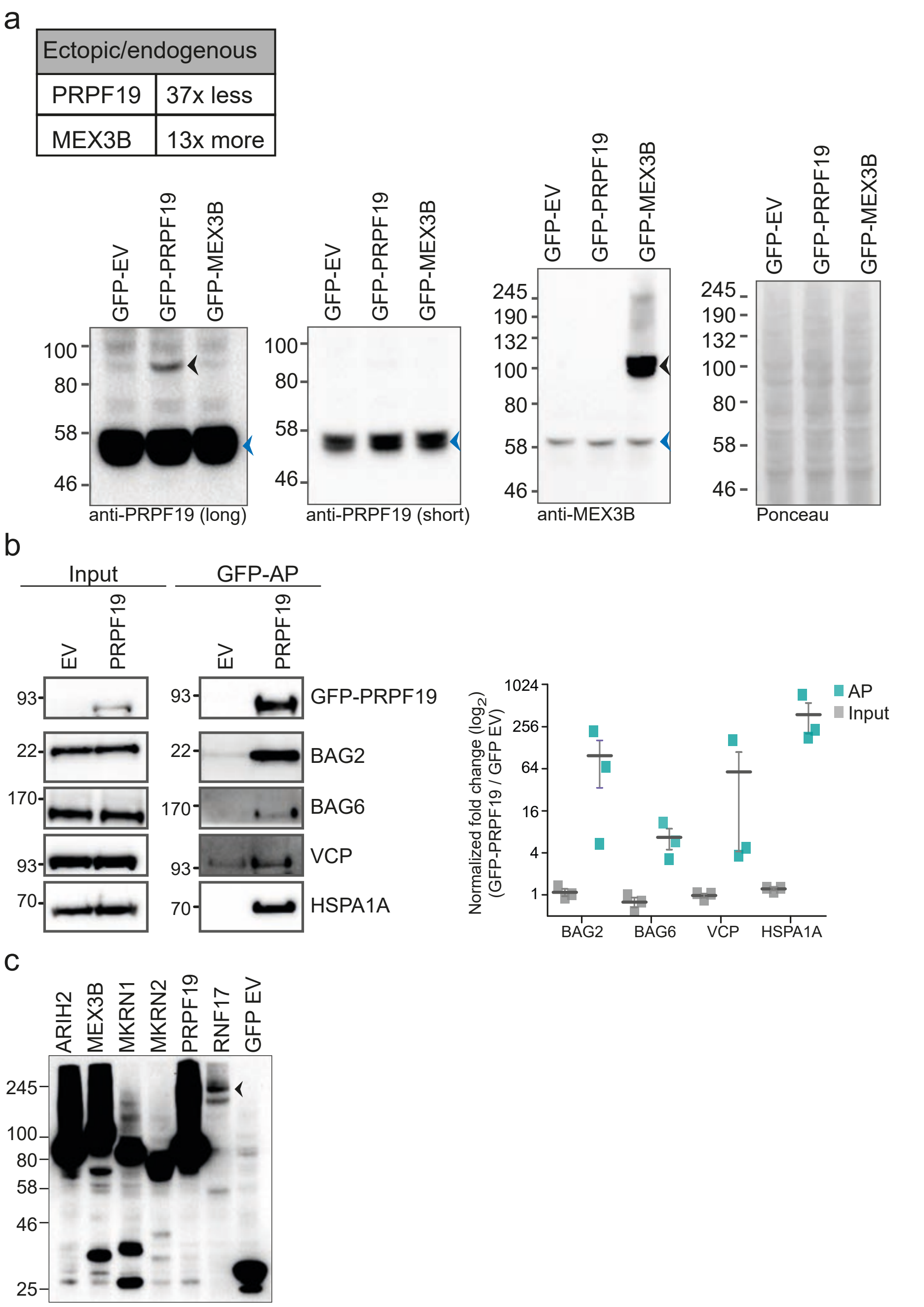

Supplementary Figure 3. Validation of RBUL interaction partners by pulldowns and Western blot (corresponding to Figure 4c, d and 2d). a. Comparison of the expression levels of GFP-tagged and endogenous PRPF19 and MEX3B. The ratios of GFP-tagged vs. endogenous PRPF19 and MEX3B are given above. Western blot analyses show the expression of GFP-PRPF19 (black arrowhead) and endogenous PRPF19 (blue arrowhead) in short and long exposure. Black and blue arrowheads indicate extrinsic GFP-PRPF19 and endogenous PRPF19, respectively. GFP-MEX3B (black arrowhead) is compared to endogenous MEX3B (blue arrowhead) by Western blot. The empty vector (EV) expressing only GFP was transfected as a control. Ponceau staining is shown as a control. The experiment was performed in two replicates (see Supplementary Figure 4j). **b.** GFP and GFP-PRPF19 were expressed in HEK293T cells and pulled down with a GFP-specific antibody. Western blot analysis was performed with antibodies against BAG2, BAG5, VCP, and HSPA1A, as well as GFP to validate the AP. Left: Cropped images of input and AP samples (replicate 1). After the GFP-specific Western blot, the membrane was cut. All membrane pieces are depicted in full size for all replicates in Supplementary Figure 4g-i. Right: Quantifications of the APs normalized to EV for three independent biological replicates are shown in a dot plot, including mean and standard error of the mean (error bars). **c.** Expression of GFP-RBULs and GFP (EV) measured by Western blot. A long exposure of the Western blot from Figure 2d is shown. GFP-RNF17 is indicated by an arrowhead.

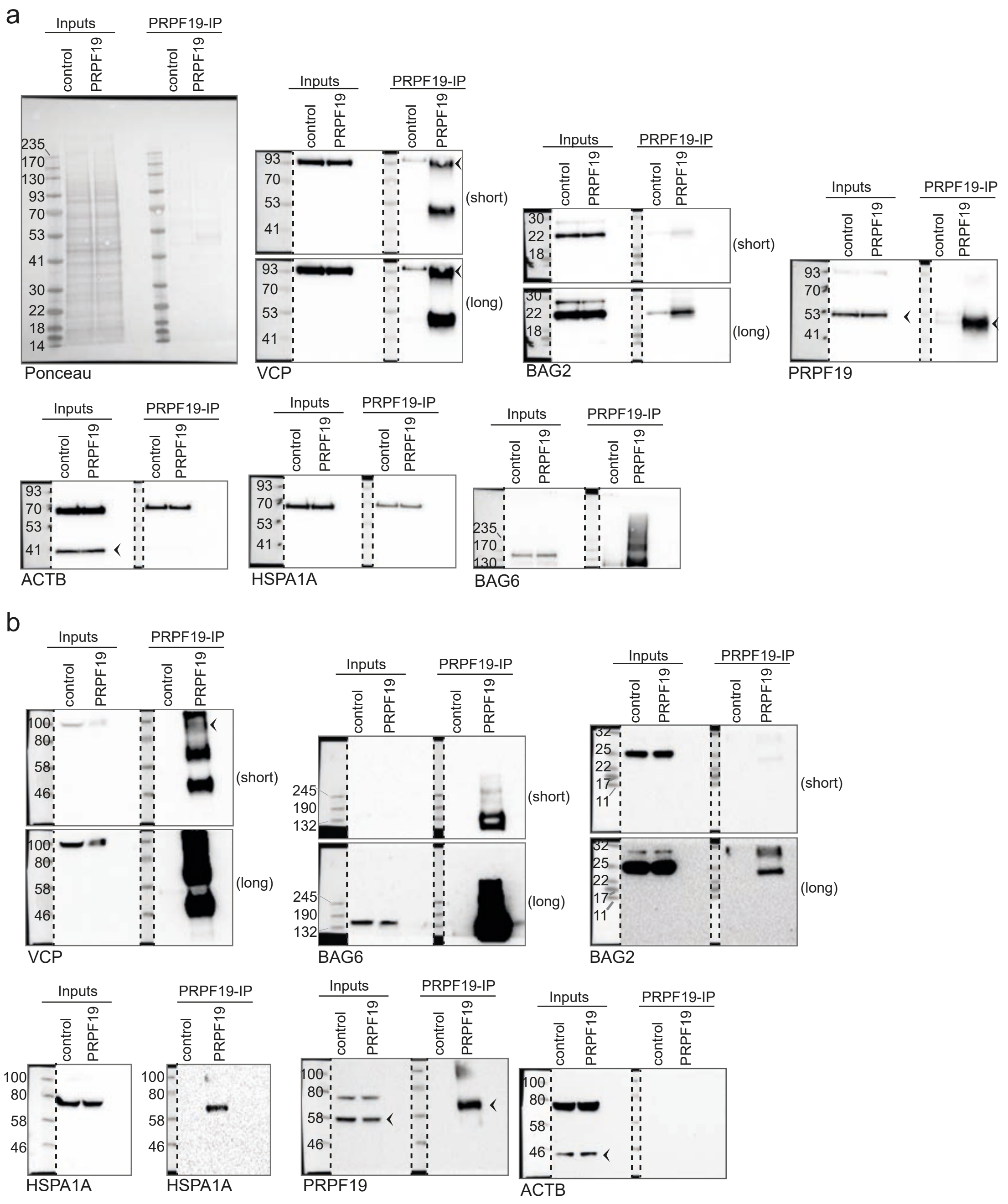
a
Inputs
PRPF19-IP
control
PRPF19
235
170
130
93
70
53
41
30
22
18
14
Ponceau
VCP
(short)
(long)
BAG2
PRPF19
ACTB
HSPA1A
BAG6
b
100
80
58
46
245
190
132
32
25
17
11
VCP
BAG6
BAG2
HSPA1A
PRPF19
ACTB

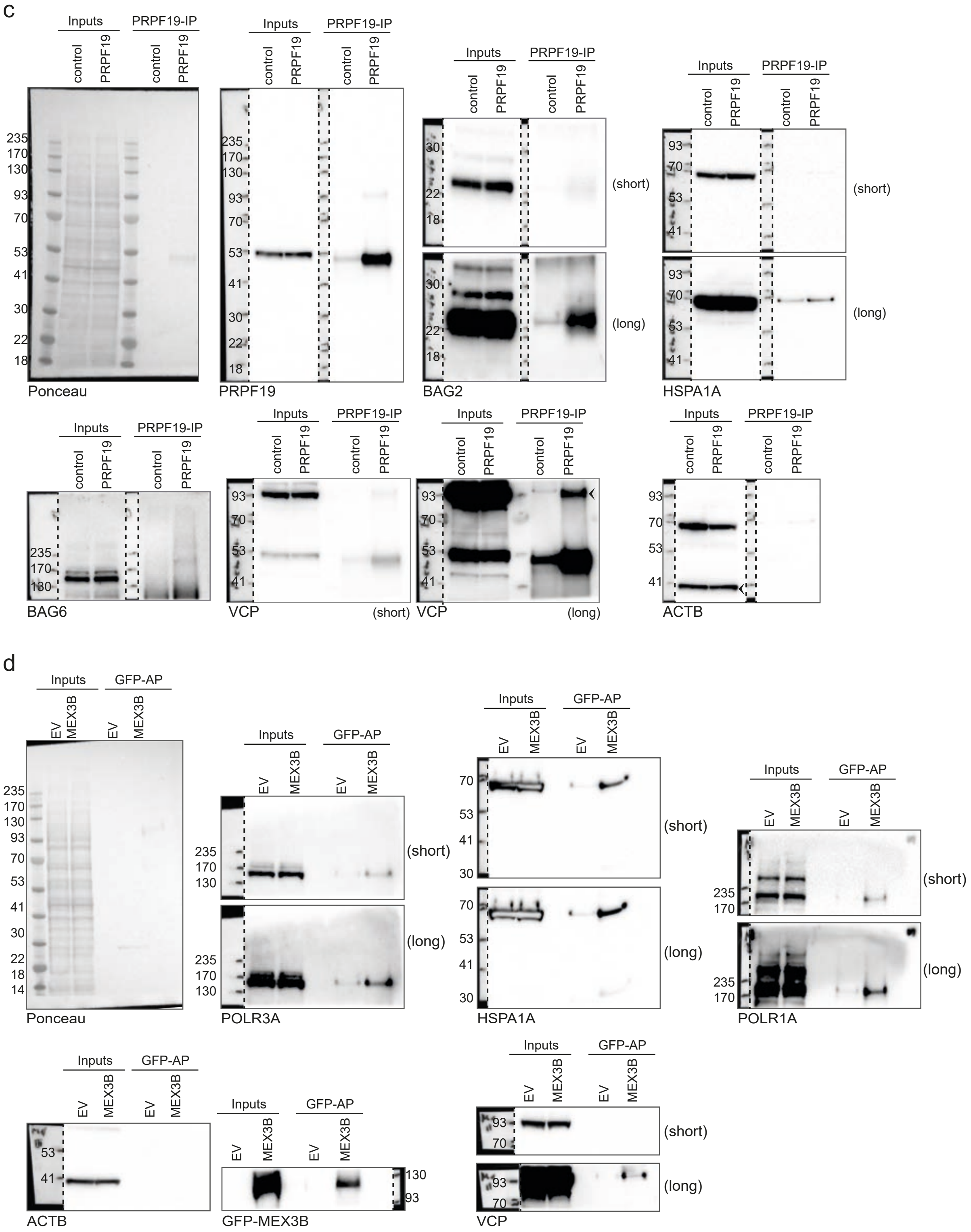
c
Inputs
PRPF19-IP
control
PRPF19
235
170
130
93
70
53
41
30
22
18
Ponceau
PRPF19
BAG2
(short)
(long)
HSPA1A
BAG6
VCP
ACTB
d
Inputs
GFP-AP
EV
MEX3B
14
Ponceau
POLR3A
HSPA1A
POLR1A
ACTB
GFP-MEX3B
VCP

e

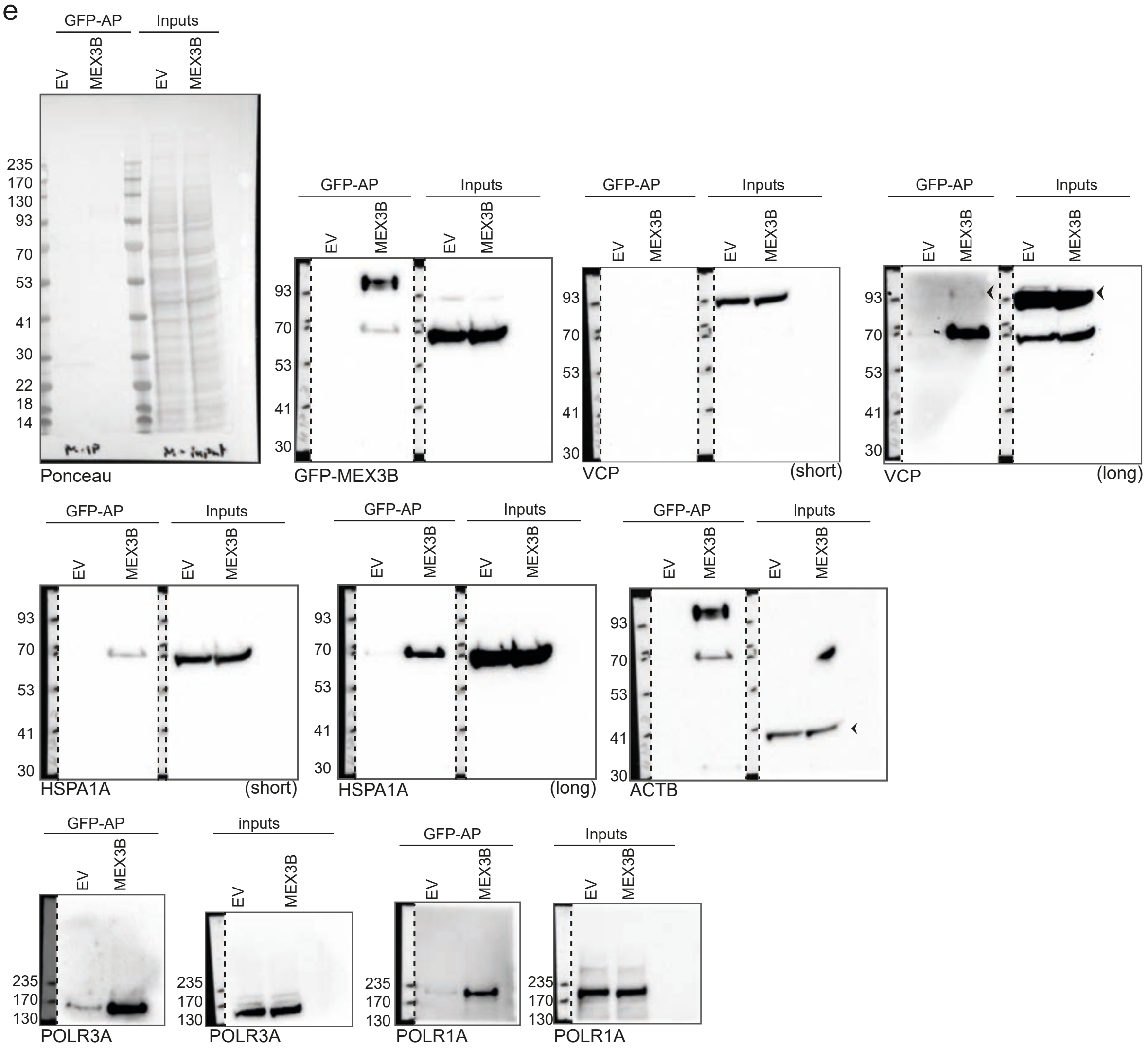
GFP-AP
Inputs
EV
MEX3B
235
170
130
93
70
53
41
30
22
18
14
Ponceau
GFP-MEX3B
VCP
(short)
VCP
(long)
HSPA1A
(short)
HSPA1A
(long)
ACTB
inputs
POLR3A
POLR1A

f

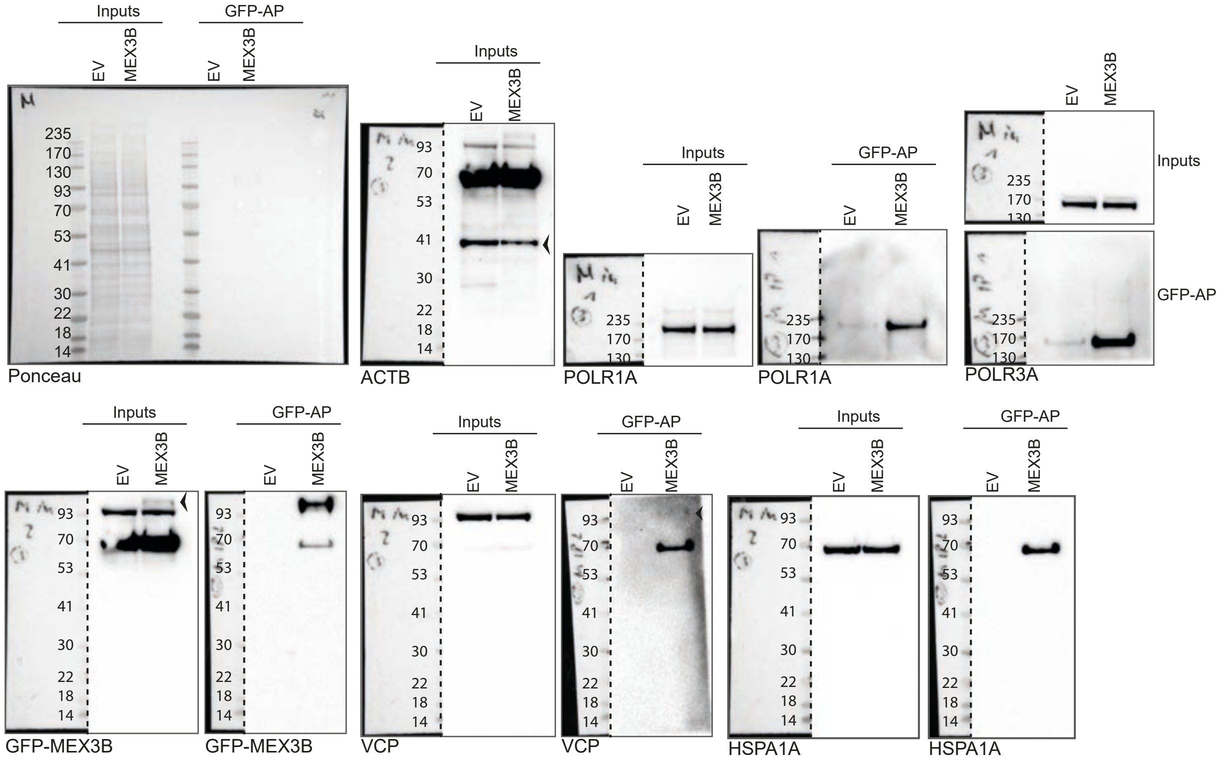
Inputs
GFP-AP
EV
MEX3B
235
170
130
93
70
53
41
30
22
18
14
Ponceau
ACTB
POLR1A
POLR3A
GFP-MEX3B
VCP
HSPA1A

g

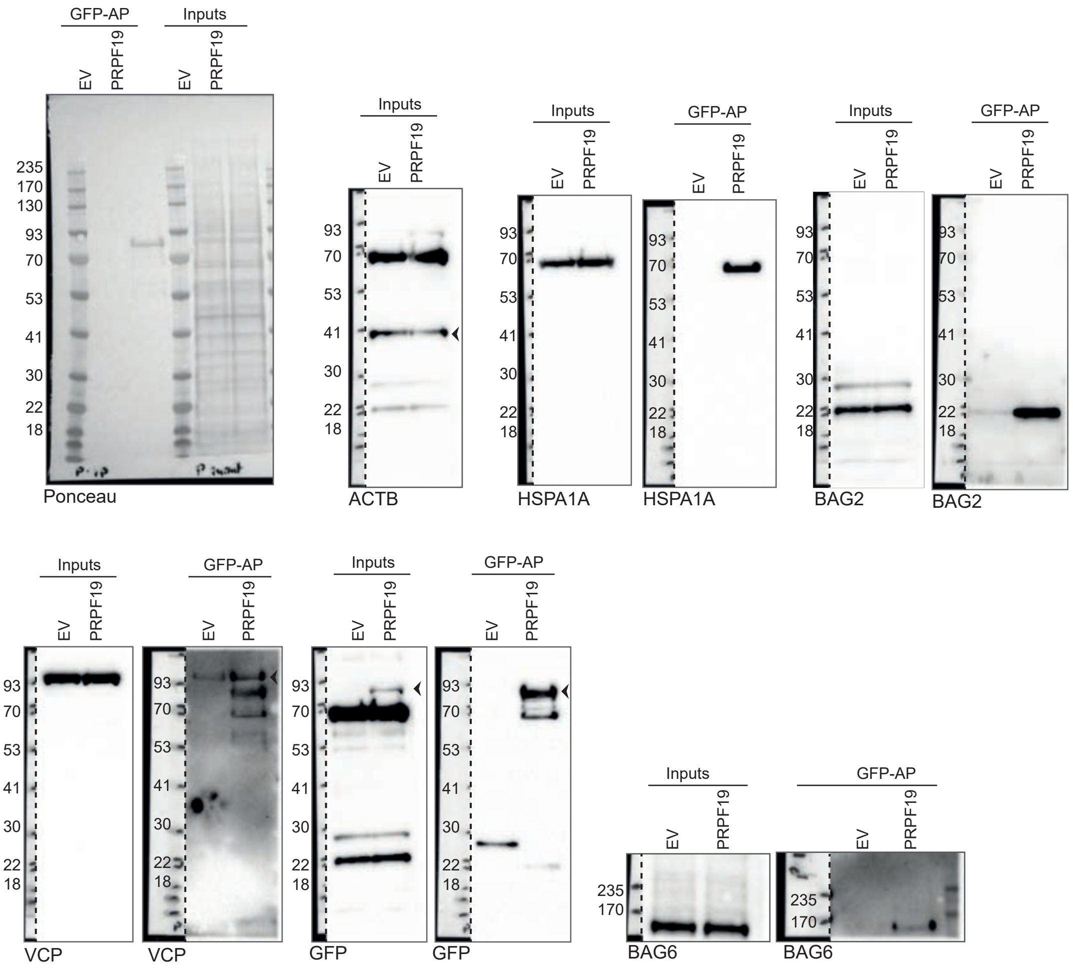
GFP-AP
Inputs
EV
PRPF19
235
170
130
93
70
53
41
30
22
18
Ponceau
ACTB
HSPA1A
BAG2
VCP
GFP
BAG6

h

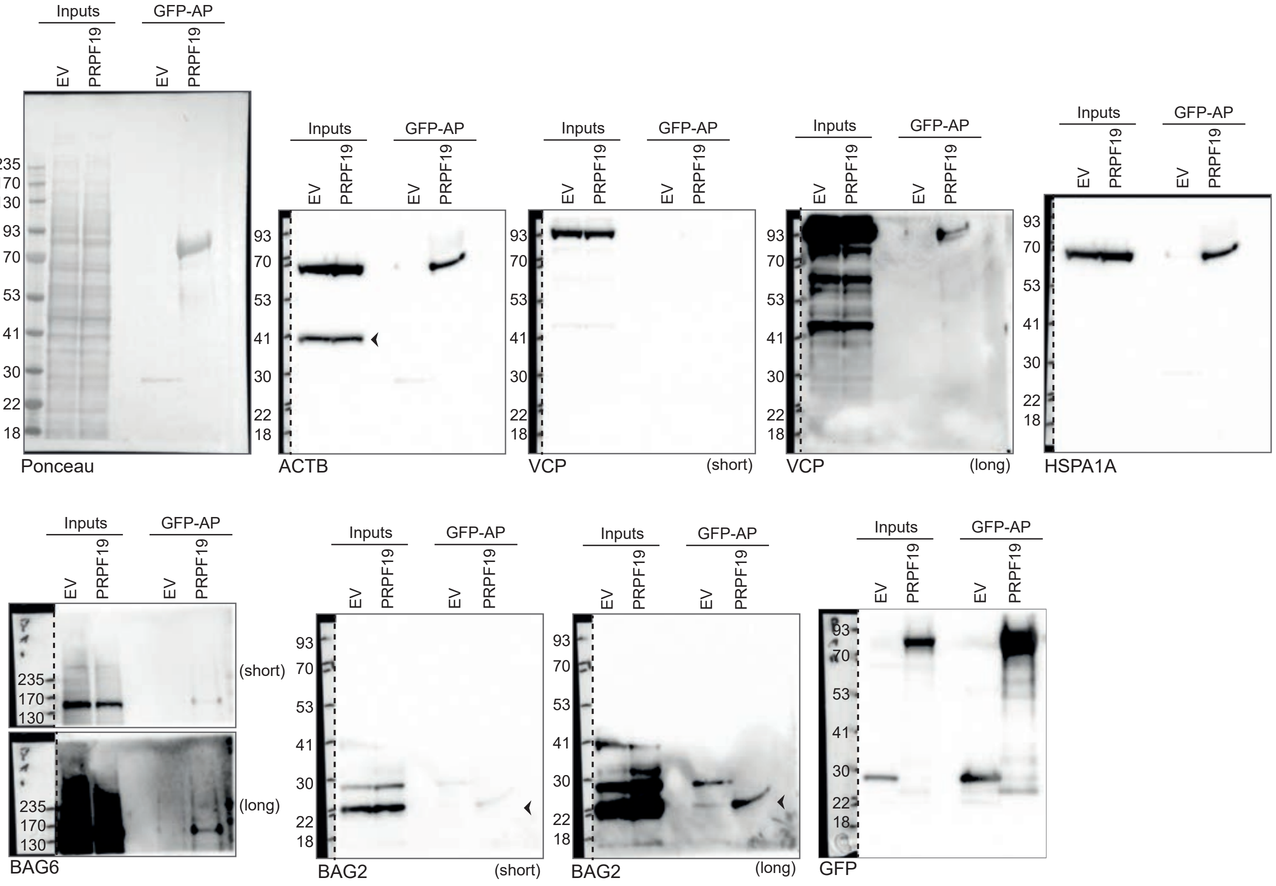
Inputs
GFP-AP
EV
PRPF19
235
170
130
93
70
53
41
30
22
18
Ponceau
ACTB
VCP
(short)
(long)
HSPA1A
BAG6
BAG2
GFP

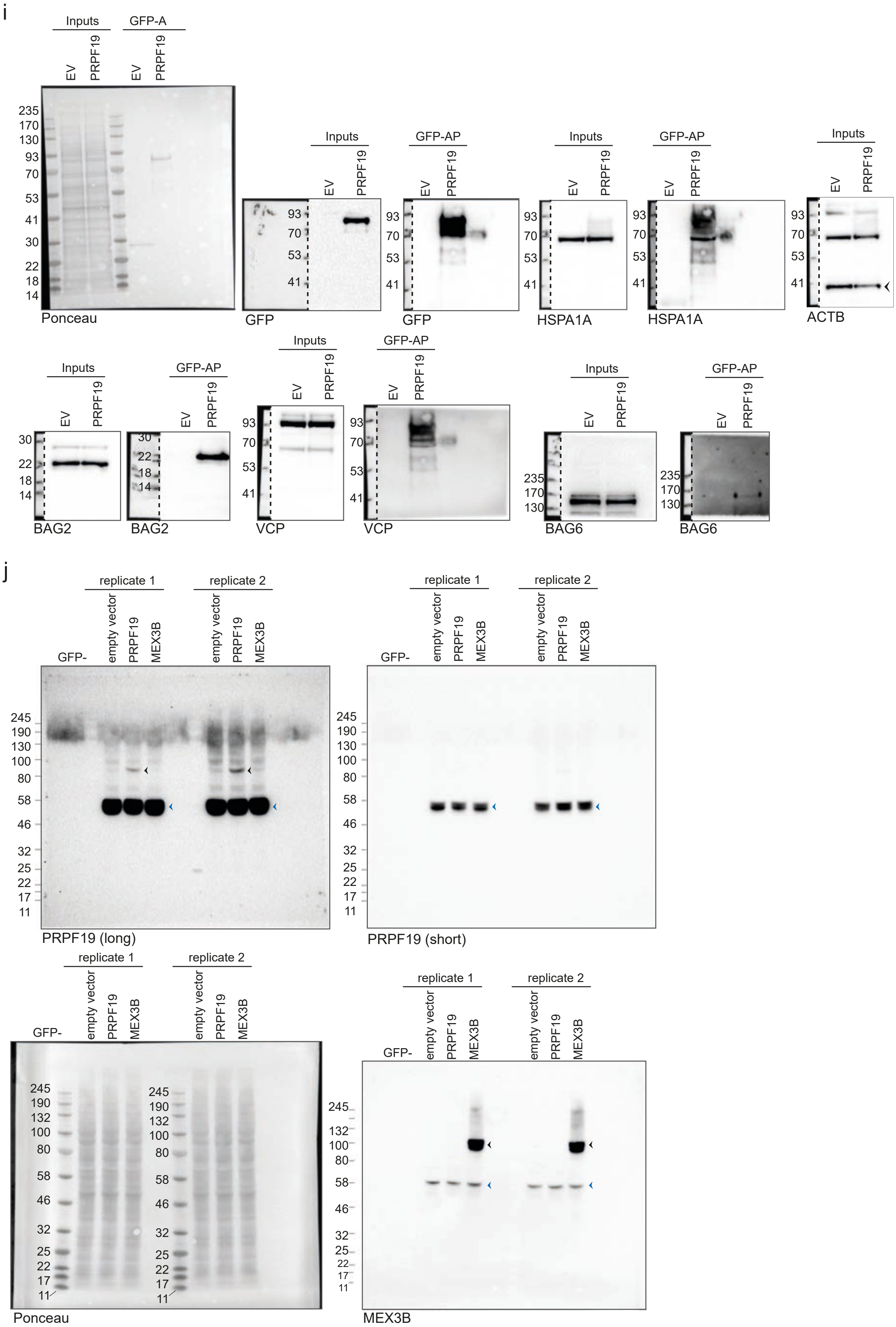
i
Inputs
GFP-A
EV
PRPF19
235
170
130
93
70
53
41
30
22
18
14
Ponceau
Inputs
GFP-AP
GFP
GFP
HSPA1A
HSPA1A
ACTB
BAG2
BAG2
VCP
VCP
BAG6
BAG6
j
replicate 1
replicate 2
GFP-
empty vector
MEX3B
245
190
130
100
80
58
46
32
25
22
17
11
PRPF19 (long)
PRPF19 (short)
132
Ponceau
MEX3B

Supplementary Figure 4. Images of full membranes and different exposure times for Western blot analyses in Figure 4c, d, Supplementary Figure 3a and b. a – c. Immunoprecipitation of endogenous PRPF19 with a PRPF19-specific antibody; extension of Figure 4c. Western blot analysis was performed with antibodies specific against BAG2, BAG6, VCP, and HSPA1A, as well as against PRPF19 itself to validate the immunoprecipitation (IP). Images of full membranes and different exposure times for all antibodies are shown for replicate 1 (a) which is presented in Figure 4c, as well as replicate 2 (b) and replicate 3 (c). **d – f.** Affinity purification of GFP (empty vector, EV) and GFP-MEX3B with a GFP-specific antibody; extension of Figure 4d. Western blot analysis was performed with antibodies specific against POLR1A, POLR3A, VCP, and HSPA1A, as well as GFP to validate the AP. Images of full membranes and different exposure times for all antibodies are shown for replicate 1 (d) which is presented in Figure 4d, as well as replicate 2 (e) and replicate 3 (f). **g – i.** Affinity purification of GFP (empty vector, EV) and GFP-PRPF19 with a GFP-specific antibody; extension of Supplementary Figure 3b. Western blot analysis was performed with antibodies specific against BAG2, BAG6, VCP, and HSPA1A, as well as GFP to validate the AP. Images of full membranes and different exposure times for all antibodies are shown for replicate 1 (g) which is presented in Supplementary Figure 3b, as well as replicate 2 (h) and replicate 3 (i). **j.** Images of full membranes for comparison of the expression levels of GFP-tagged and endogenous PRPF19 and MEX3B are shown; extension of Supplementary Figure 3a. Black arrowheads indicate the GFP-tagged RBUL, while blue arrowheads indicate the endogenous RBUL (PRPF19 or MEX3B). The empty vector (EV) expressing only GFP was transfected as a control. Ponceau staining is shown as a control. The experiment was performed in two replicates.

Supplementary Table 1. MaxQuant analysis of MS data from the conventional SILAC-AP and the adapted AP for the six RBULS. MS raw data files for the conventional SILAC-AP and the adapted AP experiments for ARIH2, MEX3B, MKRN1, MKRN2, PRPF19, and RNF17 were analyzed with MaxQuant as described in the methods section. Single tabs contain the data of the conventional SILAC-AP or the adapted AP for the respective RBUL. Protein and gene names are highlighted in light blue. Columns containing the z-score of the log-transformed GFP-RBUL vs. GFP SILAC ratios ("z-score Ratio M/L"), the z-score of the log-transformed GFPRBUL + RNase vs. GFP SILAC ratios ("z-score Ratio H/L"), and the z-score of the log-transformed GFP-RBUL + RNase vs. GFP-RBUL SILAC ratios ("z-score Ratio H/M") from the conventional SILAC-AP experiments (“RBUL_conventional_SILAC_AP”) as well as the z-scores of the log-transformed GFP-RBUL + RNase vs. GFP SILAC ratios ("z-score Ratio H/L") from the adapted AP experiments (“RBUL_adapted_ AP”) are highlighted in green.

Supplementary Table 2. Summary of adapted AP interactome analyses for the six RBULs. Gene names, protein names, annotated Biological Process (BP) GO terms, SILAC H/L ratios (z-score), Wang’s Index (WI) comparing all BPs of prey and bait, and interactions with WI ≥ 0.414 for each RBUL (ARIH2, MEX3B, MKRN1, MKRN2, PRPF19, and RNF17) are given. It is indicated if the respective interaction was considered as "TRUE" or "FALSE" considering the WI. In case of unavailability of a GO term for a prey, the WI was set to -1. Interaction partners with a z-score ≥ 2 in the adapted AP are highlighted in blue. The respective RBUL is highlighted in green.

Supplementary Table 3. Comparison of numbers of interaction partners in the conventional SILAC-AP to the adapted AP experiments. Numbers of total quantified protein groups as well as of interaction partners with a z-score ≥ 2 and detected at least in two replicates are given for the conventional SILAC-AP and the adapted AP with the six RBULs (ARIH2, MEX3B, MKRN1, MKRN2, PRPF19 and RNF17). For the conventional SILAC-AP, the number of proteins is separated into RNase-dependent and RNase-independent interactions.

	Conventional SILAC-AP			Adapted AP	
	Total # of quantified proteins	# of proteins with z-score ≥ 2, RNase-independent	# of proteins lost upon RNase-treatment	Total # of quantified proteins	# of proteins with z-score ≥ 2
ARIH2	653	14	3	963	42
MEX3B	470	6	3	795	35
MKRN1	408	5	4	572	9
MKRN2	387	12	1	717	17
PRPF19	632	11	5	1,061	91
RNF17	395	6	5	377	17

Supplementary Table 4. DAVID GO ontology enrichment for GFP-MEX3B interactors. GO enrichment analyses for 35 GFP-MEX3B interactors (together with MEX3B) were performed using DAVID. Enriched GO terms (adjusted *p*-value < 0.05) from the domains Biological Process (BP), Cellular Component (CC), and Molecular Function (MF) are listed together with the GO ID, the number, percentage and names of associated interactors (#), the total number of associated proteins in the genome ('Total') and the adjusted *p*-value (Benjamini-Hochberg correction).

GO	ID	Term	#	%	Interactors	Total	Adjusted p-value
BP	GO:0045815	Positive regulation of gene expression, epigenetic	5	13,9	POLR2H, HIST1H4A, POLR1A, H2AFY, POLR1C	62	2,75E-03
BP	GO:0043488	Regulation of mRNA stability	5	13,9	EIF4G1, PSMC5, HSPA1A, RPS27A, HSPA8	103	1,02E-02
BP	GO:0006342	Chromatin silencing	4	11,1	HIST1H2AC, H2AFV, H2AFY, HIST1H2AJ	45	1,25E-02
BP	GO:0032481	Positive regulation of type I interferon production	4	11,1	POLR2H, POLR3A, POLR1C, DHX36	51	1,36E-02
CC	GO:0000786	Nucleosome	6	16,7	HIST1H2AC, HIST1H2BC, HIST1H4A, H2AFV, H2AFY, HIST1H2AJ	94	7,70E-05
CC	GO:0070062	Extracellular exosome	18	50,0	HIST1H2AC, HIST1H2BC, PFKL, GART, PSMC5, HIST1H4A, VCP, HUWE1, TXNDC17, H2AFV, CSE1L, SQSTM1, RPL34, H2AFY, DHX36, HIST1H2AJ, HSPA8, RPS27A	2811	1,19E-04
CC	GO:0005634	Nucleus	24	66,7	POLR2H, HIST1H2AC, HIST1H2BC, MEX3B, AKAP8L, POLR1A, EIF4G1, RECQL, ZGPAT, ARIH2, PSMC5, HIST1H4A, CSE1L, H2AFV, VCP, HUWE1, DPM1, MEX3C, H2AFY, DHX36, RANBP1, HIST1H2AJ, RPS27A, HSPA8	5415	1,28E-04

(continued on next page)

(continued from previous page)

GO	ID	Term	#	%	Interactors	Total	Adjusted p-value
CC	GO:0005829	Cytosol	19	52,8	POLR2H, PFKL, POLR3A, HSPA1A, POLR1C, GART, EIF4G1, PSMC5, VCP, HUWE1, TXNDC17, CSE1L, SEH1L, SQSTM1, RPL34, DHX36, TNRC6A, RPS27A, HSPA8	3315	1,60E-04
CC	GO:0005654	Nucleoplasm	17	47,2	POLR2H, HIST1H2BC, AKAP8L, POLR1A, POLR3A, HSPA1A, POLR1C, RECQL, PSMC5, VCP, HUWE1, CSE1L, HIST1H4A, SQSTM1, TNRC6A, HSPA8, RPS27A	2784	2,24E-04
CC	GO:0005736	DNA-directed RNA polymerase I complex	3	8,3	POLR2H, POLR1A, POLR1C	13	4,11E-03
CC	GO:0005666	DNA-directed RNA polymerase III complex	3	8,3	POLR2H, POLR3A, POLR1C	19	6,70E-03
CC	GO:0016234	Inclusion body	3	8,3	PSMC5, SQSTM1, HSPA1A	18	6,86E-03
CC	GO:0005737	Cytoplasm	19	52,8	HIST1H2BC, PFKL, AKAP8L, POLR1A, HSPA1A, GART, EIF4G1, RECQL, ARIH2, PSMC5, VCP, HUWE1, CSE1L, SQSTM1, RPL34, MEX3C, DHX36, RANBP1, RPS27A	5222	2,57E-02
CC	GO:0005913	Cell-cell adherens junction	5	13,9	EIF4G1, RPL34, RANBP1, HSPA1A, HSPA8	323	2,77E-02
CC	GO:0000790	Nuclear chromatin	4	11,1	HIST1H2AC, H2AFV, H2AFY, HIST1H2AJ	193	4,27E-02
CC	GO:0016020	Membrane	11	30,6	EIF4G1, RECQL, PSMC5, HIST1H4A, CSE1L, PFKL, HUWE1, DPM1, POLR3A, RPS27A, HSPA8	2200	4,62E-02

(continued on next page)

(continued from previous page)

GO	ID	Term	#	%	Interactors	Total	Adjusted p-value
MF	GO:0003677	DNA binding	13	36,1	POLR2H, HIST1H2AC, HIST1H2BC, POLR1A, AKAP8L, POLR3A, POLR1C, RECQL, HUWE1, H2AFV, HIST1H4A, H2AFY, HIST1H2AJ	1674	3,24E-03
MF	GO:0044822	poly(A) RNA binding	11	30,6	EIF4G1, HIST1H4A, HUWE1, VCP, MEX3B, MEX3C, AKAP8L, DHX36, TNRC6A, RPS27A, HSPA8	1129	3,98E-03
MF	GO:0003899	DNA-directed RNA polymerase activity	4	11,1	POLR2H, POLR1A, POLR3A, POLR1C	38	7,70E-03
MF	GO:0001054	RNA polymerase I activity	3	8,3	POLR2H, POLR1A, POLR1C	12	7,99E-03
MF	GO:0001056	RNA polymerase III activity	3	8,3	POLR2H, POLR3A, POLR1C	18	1,47E-02

Supplementary Table 5. Known and novel protein-protein interactions identified from the adapted APs of the six RBULs. A comparison of identified interaction partners from the adapted AP to known interactions from the literature is shown. Out of 170 interactions in total, only 19 were reported in literature to date. For each RBUL, the number of interactions reported in HIPPIE together their HIPPIE confidence score and the kind of experiment used to measure in combination with PubMed identifiers (PMIDs) and reporting databases are given. All remaining interactors not present in HIPPIE are listed as a block below.

Bait	HIPPIE inter-actions	Interactor	Score	Sources
ARIH2	4	CUL5	0.78	enzymatic study, affinity chromatography technology , pull down, anti tag coimmunoprecipitation, anti bait coimmunoprecipitation (PMID: 24076655; sources: BioGRID, IntAct)
		TCEB1	0.72	affinity chromatography technology, anti tag coimmunoprecipitation (PMIDs: 24076655; sources: BioGRID, IntAct)
		HK2	0.63	affinity chromatography technology (PMID: 22863883; source: BioGRID)
		VCP	0.63	affinity chromatography technology (PMID: 23383273; source: BioGRID)
	Not present	ZC3H7A, RNF20, PRPSAP2, PPM1G, DDX1, GSTO1, PAPSS1, CHERP, Nbla03646, MID1, GNE, POLR3A, APPL1, GTF2H2, PRKCI, PYGB, DOCK7, ASNS, ALDH2, TUBB4A, CAPN1, CAPN2, TRMT1, RPS27A, PGM1, RFC5, ABCE1, SRP54, ZGPAT, EIF1AX, ACTR2, BCKDK, GANAB, RPL34, UROD, ASCC3, DHX15, MEX3B		
MEX3B	Not present	RPS27A, MEX3C, SQSTM1, HSPA1A, HSPA8, POLR1A, POLR2H, PDE12, PFKL, H2AFV, POLR1C, HIST1H4A, HIST1H2BC, HUWE1, EIF4G1, HIST1H2AJ, DHX36, CSE1L, H2AFY, DPM1, AKAP8L, HIST1H2AC, PSMC5, TXNDC17, RECQL, METTL13, GART, RANBP1, SEH1L, TNRC6A, RPL34, ZGPAT, POLR3A, VCP		
MKRN1	Not present	VCP, RPS27A, HSPA1A, IGSF9B, RPS27L, EXD2, ZNF512, C3orf17, UBAP2L, VCP, RPS27A		
MKRN2	Not present	RPS27A, VCP, RPL34, IGSF9B, PABPC4, PABPC1, CHTOP, SET, GOLGA4, ST13, RPS12, RPL37A, YWHAZ, ATP2B1, MSH3, HSPA1A, HSPA8		

(continued on next page)

(continued from previous page)

Bait	HIPPIE inter-actions	Interactor	Score	Sources
PRPF19	17	CDC5L	0,9	affinity Capture-MS, copurification, pull down, affinity chromatography technology, anti bait coimmuno-precipitation, far western blotting (PMIDs: 11101529, 20467437, 19633697, 20595234, 20176811, 17276391, 22939629, 26344197; sources: BioGRID, MINT, IntAct)
		BCAS2	0.89	two-hybrid, anti tag coimmuno-precipitation, affinity chromatography technology, far western blotting, pull down (PMIDs: 22365833, 20176811, 21536736, 22939629, 26344197; sources: MINT, BioGRID, IntAct)
		PLRG1	0.87	affinity Capture-MS, anti bait co-immunoprecipitation, affinity chroma-tography technology (PMIDs: 17353931, 20176811, 17276391, 26344197; sources: BioGRID, IntAct, I2D)
		PRPF8	0,87	two-hybrid, anti tag coimmuno-precipitation, affinity chromatography technology (PMIDs: 22365833, 22939629, 26344197; sources: MINT, IntAct, BioGRID)
		CDC40	0.76	two-hybrid, anti tag coimmuno-precipitation, affinity chromatography technology (PMID: 22365833; sources: MINT, IntAct, BioGRID)
		SNW1	0.73	anti bait coimmunoprecipitation (PMIDs: :20467437, 26344197; sources: MINT, IntAct, BioGRID)
		HSP90AA1	0.63	affinity chromatography technology (PMID: 22939624; source: BioGRID)
		HUWE1	0.63	affinity chromatography technology (PMID: 25147182; source: BioGRID)
		VCP	0,63	affinity chromatography technology (PMID: 23443559; source: BioGRID)
		SF3B3	0.59	PMIDs: 22939629, 26344197; source: BioGRID
		SNRNP200	0,59	PMIDs: 22939629, 26344197; source: BioGRID
		XAB2	0.59	PMIDs: 22939629, 26344197; source: BioGRID

(continued on next page)

(continued from previous page)

Bait	HIPPIE inter-actions	Interactor	Score	Sources
PRPF19		AQR	0.55	cosedimentation through density gradient (PMID: 24304693; source: IntAct)
		DHX8	0.49	PMIDs: 26344197; source: BioGRID
		EFTUD2	0,49	PMID: 22939629; source: BioGRID
		HSP90AB1	0,49	PMID: 22939624; source: IntAct
		RNPS1	0,49	PMID: 22939629; source: BioGRID
	Not present	HIST1H4A, HIST1H2BC, EIF4G1, HIST1H2AJ, H2AFY, HIST1H2AC, IGSF9B, CHTOP, PFDN4, UBL4A, VBP1, PFDN1, BAT3, PFDN2, CCT2, ASNA1, PFDN6, BAG2, CCT8, CCT5, CCT6A, GET4, CCT4, CCT7, CCT3, TCP1, HSPA6, TAB1, SPAG9, RHOT2, PSMD2, SUGT1, RPAP3, NACA, PSMC6, RBM22, CRNKL1, EPB41L2, DDB1, DNAJB6, USP19, RBM8A, FBL, POLR2B, DNAJC7, DNAJA1, PSMC4, SNRNP40, HNRNPC, TP53BP2, ZNF326, SNRPD3, TBC1D4, IRS4, TCOF1, TRA2A, PNN, DNAJA2, PSMC1, PSMC2, STIP1, MDC1, H1F0, GNB2L1, FTSJ3, RSL1D1, RBMX, SF3B2, SNRPE, SNRPN, RNF17, HSPA8, HSPA1A, RPS27A, POLR3A		
RNF17	Not present	VCP, RPS27A, HSPA1A, CCT2, BAG2, CCT8, CCT6A, CCT4, HSPA6, HSPA8, GOLGA4, ACIN1, SSBP1, PGAM5, RAB1B, RPLP2		

Supplementary Table 6. Summary of the cellular localization of the RBULs. The cellular localization according to the Human Protein Atlas (www.proteinatlas.org), as well as the observed cellular localization in our microscopy experiments ("Cellular localization - our data") are given for each RBUL. PubMed identifier (PMIDs) for further localization studies are mentioned in the "Additional references" column.

RBUL	Cellular localization - Human Protein Atlas	Cellular localization - our data	Additional references
ARIH2	Nucleoplasm	Nucleoplasm and cytosol	PMIDs: 25613900, 21139605, 18669619, 16127175, 11435423, 19340006
MEX3B	Nucleoplasm and cytosol (shuttling); cytosol and cytosolic granules	Nucleoplasm periphery, cytosol, and cytosolic granules	PMIDs: 25613900, 21139605, 18669619, 16127175, 11435423, 17267406, 18779327
MKRN1	Nucleus, nuclear membrane, and cytosol	Nucleus, cytosol, and cytosolic granules	PMIDs: 25613900, 21139605, 18669619, 16127175, 11435423, 22128154
MKRN2	Nucleoplasm and cytosol	Cytosol	PMIDs: 25613900, 21139605, 18669619, 16127175, 11435423
PRPF19	Nuclear speckles	Nucleoplasm and cytosol	PMIDs: 25613900, 21139605, 18669619, 16127175, 11435423, 20176811
RNF17	No data	Cytosol and cytosolic granules	PMIDs: 25613900, 21139605, 18669619, 16127175, 11435423
